# Development of the Breastfed Infant Oral Microbiome Is Associated with Concentrations and Intakes of Human Milk Oligosaccharides

**DOI:** 10.3390/nu17223622

**Published:** 2025-11-20

**Authors:** Roaa A. Arishi, Ali S. Cheema, Jacki L. McEachran, Zoya Gridneva, Annalee Furst, Ana Roman, Lars Bode, Ching Tat Lai, Matthew S. Payne, Donna T. Geddes, Lisa F. Stinson

**Affiliations:** 1 School of Molecular Sciences, The University of Western Australia, Crawley, WA 6009, Australia; roaa.arishi@research.uwa.edu.au (R.A.A.); zoya.gridneva@uwa.edu.au (Z.G.); donna.geddes@uwa.edu.au (D.T.G.); 2 ABREAST Network, Perth, WA 6000, Australia; 3 UWA Centre for Human Lactation Research and Translation, Crawley, WA 6009, Australia; 4 Ministry of Education, Riyadh 14811, Saudi Arabia; 5 The Kids Research Institute Australia, Nedlands, WA 6009, Australia; 6 Department of Pediatrics, Larsson-Rosenquist Foundation Mother-Milk-Infant Centre of Research Excellence (MOMI CORE), and the Human Milk Institute (HMI), University of California San Diego, La Jolla, CA 92093, USA; 7 Division of Obstetrics and Gynaecology, The University of Western Australia, Crawley, WA 6009, Australia

**Keywords:** infant oral microbiome, human milk, breastfeeding, human milk oligosaccharides

## Abstract

**Background/Objectives**: Human milk oligosaccharides (HMOs) are bioactive carbohydrates abundant in human milk that shape the infant gut microbiome, yet their influence on the oral microbiome remains poorly understood. This study investigated associations between HMO concentrations and infant HMO intakes and the composition of the oral microbiome in predominantly (*n* = 2) and exclusively (*n* = 54) breastfed infants. **Methods**: We profiled infant oral samples collected at 2 months of age using full-length 16S rRNA gene sequencing, alongside paired milk HMO analyses from 56 mother–infant dyads from the Western Australian BLOSOM cohort. Daily HMO intakes were calculated using 24 h milk intake data. **Results:** Concentrations of human milk 3FL and LNFPIII were negatively associated with infant oral Shannon diversity (*p* = 0.027) and richness (*p* = 0.037), respectively. LNFPII concentration and daily intake were linked to increased abundance of *Neisseria subflava* (both *p* = 0.025), while daily intakes of DFLNT and DFLNH were positively associated with *Streptococcus parasanguinis* (*p* = 0.028 and *p* = 0.040). Notably, the associations observed for daily HMO intakes were modest in effect size. **Conclusions:** These findings demonstrate a statistically significant but biologically limited effect of HMOs on the development of the infant oral microbiome. By clarifying how specific components of human milk shape early microbial development, this work provides mechanistic insights relevant to nutritional and supportive interventions that promote breastfeeding success.

## 1. Introduction

The first six months of life represent a critical window for the establishment of the infant oral microbiome, during which breastfeeding supports the colonisation of beneficial bacteria [1,2,3]. Among its many bioactive components, human milk oligosaccharides (HMOs) are the most abundant, with concentrations of approximately 20–25 g/L in colostrum and 5–20 g/L in mature milk [4]. These structurally diverse glycans are not digested by the infant, and instead act as selective prebiotics that support the growth of beneficial gut bacteria, regulate neonatal immunity by modulating epithelial and immune cell activity in the infant gut [5], and inhibit the colonisation of intestinal epithelial surfaces by pathogenic microorganisms, thereby reducing the risk of infection in early life [6]. While HMOs are well studied in the context of gut microbial development [7,8,9,10], their impact on the infant oral microbiome remains largely understudied, with only one small study of 10 infants published to date [11].

The breastfed infant’s oral cavity is repeatedly exposed to human milk, facilitating direct interaction between HMOs and the developing oral microbiome. However, both HMO concentrations and total intake should be considered, as inter-individual variability in milk volume may result in infants receiving different total doses despite similar concentrations. The concentration of HMOs in milk may influence the infant’s oral microbiome by modulating microbial adhesion and immediate ecological interactions during feeding, whereas the total daily dose may reflect systemic effects of HMOs on the infant’s immune system [4,12].

**Figure 1:**
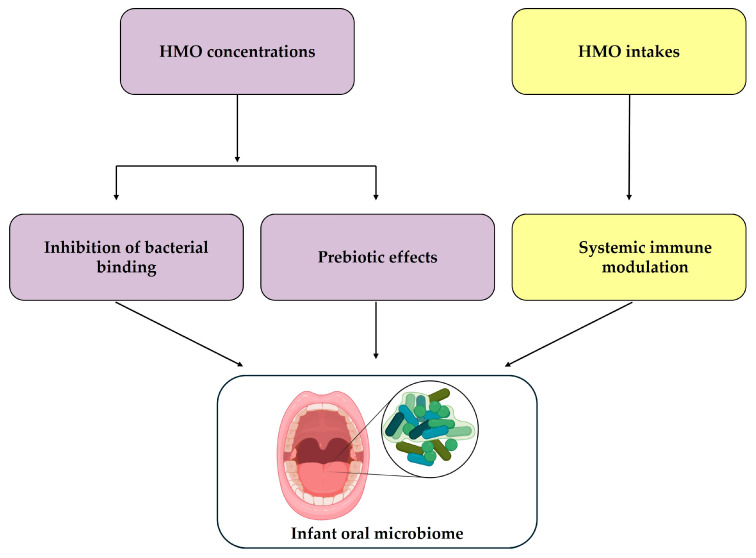
Conceptual diagram illustrating the hypothesised mechanisms through which HMOs may influence the infant oral microbial ecology. Purple boxes represent local effects driven by the concentration of HMOs in milk, while yellow boxes represent systemic effects driven by total intake of HMOs. Created in BioRender: https://BioRender.com/6901bjv (accessed on 9 November 2025).

The present study builds on the limited existing literature by examining both HMO concentrations and infant intake in relation to the oral microbiome in breastfed infants. We hypothesised that HMO concentrations and daily intakes would elicit differential effects on the infant oral microbiome due to their differing modes of action (Figure 1). To test this, we assessed associations between HMO concentrations and daily intakes and the oral microbiota of healthy, term, exclusively or predominantly breastfed infants.

## 2. Materials and Methods

### 2.1. Study Design

In this sub-study, a total of 56 mother–infant pairs with infant oral microbiome data and HMO data available were selected from the BLOSOM cohort (Breastfeeding Longitudinal Observational Study of Mothers and kids). All mothers were non-smokers, delivered full-term infants with no major health complications, and intended to breastfeed for the first year. Full cohort recruitment has been previously reported elsewhere [13]. Participants provided informed written consent, and the study received approval from the Human Research Ethics Committee of The University of Western Australia (RA/4/20/4023) on 7 March 2018.

### 2.2. Sample and Data Collection

Infant oral swabs and human milk samples were collected at 2 months postpartum. Sample collection protocols have been reported elsewhere [13]. Samples were refrigerated at home for up to 18 h and subsequently transported on ice to the laboratory, where they were immediately aliquoted into sterile tubes and stored at −80 °C until subsequent analysis. Participants provided infant feeding data at the time of sample collection.

### 2.3. Human Milk Oligosaccharides

HMO concentrations were measured by the Bode Lab at the University of California, San Diego, CA, USA, using high-performance liquid chromatography (HPLC), as previously described (25). The 19 most abundant HMOs were identified and quantified: 2′-fucosyllactose (2′FL), 3-fucosyllactose (3FL), 3′-sialyllactose (3′SL), 6′-fucosyllactose (6′FL), difucosyllactose (DFLac), difucosyl-N-hexosyl (DFLNH), difucosyl-N-acetyl (DFLNT), disialyl-N-hexosyl (DSLNH), disialyl-N-acetyl (DSLNT), fucosyl-disialyl-N-hexosyl (FDSLNH), fucosyllacto-N-hexaose (FLNH), lacto-N-fucopentaose I (LNFP I), lacto-N-fucopentaose II (LNFP II), lacto-N-fucopentaose III (LNFP III), lacto-N-hexaose (LNH), lacto-N-neotetraose (LNnT), lacto-N-tetraose (LNT), lacto-N-neohexaose (LSTb), and lacto-N-neotriose (LSTc). Maternal secretor status was determined based on the abundance of 2′FL.

### 2.4. 24 h Milk Intake

Infant milk intake was assessed at the 3-month time point using the 24 h milk profile protocol as previously described [14]. Briefly, mothers weighed their infants before and after each feed over a 24 h period using calibrated electronic infant scales (±2 g; Electronic Baby Weigh Scale, Medela Inc., McHenry, IL, USA). Human milk intake, in grams, was calculated by subtracting the pre-feed weight from the post-feed weight. Intake volume was converted from grams to millilitres using the density of human milk (1.03 g/mL) [15]. 24 h milk intake at three months was considered representative of 2-month intake, as infant milk intake does not significantly vary from one to six months [16]. The 24 h milk intake data were available for 46 of the 56 participants.

### 2.5. Daily Intake of Human Milk Oligosaccharides

Daily intakes of HMOs (µg) were calculated by multiplying the concentration of each HMO (µg/mL) by the volume of milk intake per 24 h.

### 2.6. Infant Oral Microbiome Analysis

DNA extraction from the cell pellet was carried out using the QIAGEN MagAttract Microbial DNA Isolation Kit (QIAGEN, Hilden, Germany) as previously described [13]. Amplification of the full-length 16S rRNA gene was performed with asymmetrically barcoded primers 27F and 1492R. All PCR reagents were decontaminated using the ArcticZymes PCR Decontamination Kit (ArcticZymes, Tromsø, Norway) prior to use. PCR was conducted as previously described [13], and amplicons were normalised, pooled, and purified using a Macherey Nagel NucleoMag^®^ NGS Clean-up and Size Select kit (Macherey Nagel, Düren, Germany). Purified amplicon libraries were sequenced on a PacBio Sequel II platform at the Australian Genome Research Facility (AGRF).

### 2.7. Sequencing Data Processing

Mothur v.1.48.0 was used to process full-length 16S rRNA gene sequences [17]. Alignment to the SILVA reference alignment v132 was performed after raw sequences were filtered for length (1336–1743 bp) and homopolymer content (≤9 bases) [18]. Chimeric sequences were eliminated using VSEARCH (v2.13.3) [19]. SILVA taxonomy database (v132) was used to classify the sequences [18] using a confidence threshold of 80. Sequences were grouped into operational taxonomic units (OTUs).

After excluding non-bacterial sequences. After initial classification to genus level using SILVA, BLAST (v2.17.0) [20] was used to further classify highly abundant OTUs (those with an average relative abundance > 0.5% and a prevalence of >10%; *n* = 15) to the species level, with a cutoff of 97% sequence identity and 99% sequence coverage [21] (Supplementary Table S1). In cases where multiple species mapped equally well to an OTU, genus-level classification is provided. Sequences detected in the negative extraction and template controls are presented in Supplementary Table S2.

### 2.8. Data Analysis

Sub-sampled data were used for all diversity analyses. Sub-sampling to the size of the smallest library (3747 reads) was conducted, resulting in an average sequence coverage of 86.2%. Alpha diversity was evaluated using OTU-level richness and Shannon diversity indices, while beta diversity was determined based on Bray–Curtis dissimilarity. OTU abundance data were centre log ratio (CLR)-transformed prior to analysis. All statistical analyses were conducted using R software [22]. To investigate the impact of HMO concentrations/intakes on the infant oral microbiome at two months of age, we performed linear modelling using the lm R package (v4.3.0) [22]. Models were fitted separately for each of the 15 OTUs investigated, in addition to Shannon diversity and richness. The general model formula was:

Outcome∼HMO concentration/intake+mode of delivery+siblings+maternal pre-pregnancy BMI+pacifier use during the first week of life+secretor status. Where the outcome variable was either Shannon diversity, richness, and CLR-transformed abundance of each OTU, and the independent variable of interest was the concentration or daily intake of each HMO.

Model assumptions were assessed prior to interpretation. Normality of residuals was evaluated using the Shapiro–Wilk test, and independence was satisfied as all data were collected at a single time point for each participant (Supplementary Table S3).

Variance inflation factors (VIFs) were calculated to assess multicollinearity among predictors. Several HMOs exhibited VIFs > 10, reflecting their expected intercorrelations in human milk. Given the exploratory nature of the analysis, all predictors were retained, and results were interpreted cautiously with emphasis on effect sizes and confidence intervals rather than statistical significance alone (Supplementary Table S4).

Data were pre-processed and managed using the dplyr package (v1.1.2) [23], including filtering, merging, and transforming variables as required. Visualisation was performed using ggplot2 (v4.0.1) [24]. For each model, effect sizes and 95% confidence intervals were reported alongside *p*-values to enhance interpretability.

To account for potential confounding, analyses were adjusted for mode of delivery, presence of siblings, maternal pre-pregnancy BMI, and pacifier use during the first week of life. These covariates were selected based on our previous findings demonstrating their significant associations with infant oral microbiome beta diversity in this cohort [25]. Additionally, secretor status was included as a covariate for all models. Two types of models were employed: one for concentrations of each of the 19 HMOs, and one for daily intakes of each of these HMOs. HMO intake analysis was restricted to the 46 mother–infant pairs for whom 24 h milk intake data were available.

Associations between HMO concentrations/intakes and the structure of the infant oral microbiome were assessed by PERMANOVA, implemented with the adonis2 function in the vegan package (v2.6.4) [26]. Backwards model selection was used with 999 permutations.

To account for multiple comparisons, all *p*-values were adjusted using the Benjamini–Hochberg (BH) correction method within each individual model (i.e., separately for each OTU) [27]. Adjusted *p*-values are presented throughout the text, and unadjusted values are reported in Supplementary Tables (Supplementary Tables S5–S7).

## 3. Results

All infants were born at term, had no major health complications, and were predominantly (*n* = 2) or exclusively (*n* = 54) breastfed, with an average daily milk intake of 766.9 mL (Table 1).

### 3.1. Infant Oral Microbiome Composition

**Figure 2:**
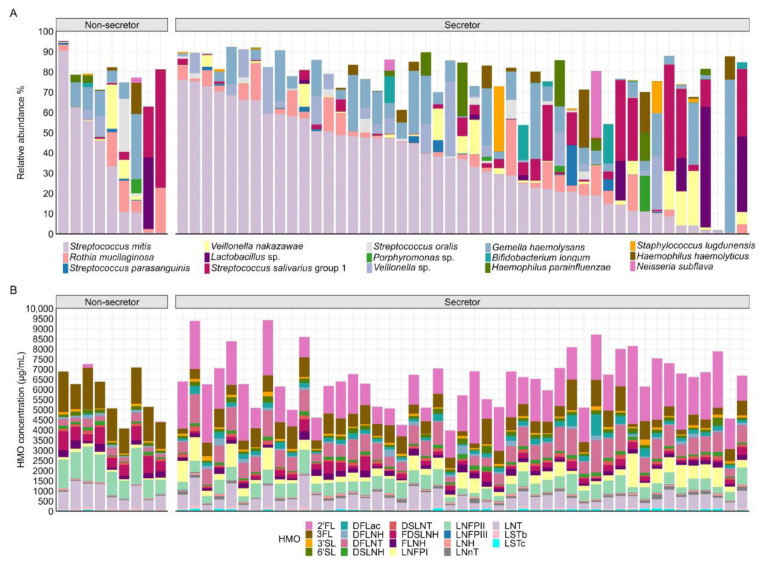
Infant oral and human milk HMO profiles across the cohort. Individuals are plotted on the x-axis, with microbiome and HMO data aligned vertically for each individual. (**A**), Relative abundances of the 15 most abundant operational taxonomic units (OTUs) in infant oral samples, stratified by maternal secretor status. (**B**), HMO concentrations in individual mothers based on secretor status. HMO, human milk oligosaccharides.

We identified 15 OTUs with an average relative abundance of ≥0.5% and a prevalence of >10%, making up a total of 77.8% of the bacterial community across all samples (Figure 2A). The most abundant OTUs in the infant oral microbiome at 2 months of age mapped to *Streptococcus mitis* (mean 34.5%), *Gemella haemolysans* (10.0%), *Rothia mucilaginosa* (6.3%), and *Streptococcus salivarius* group 1 (8.5%).

### 3.2. Human Milk Oligosaccharides

The most abundant HMOs were 2′FL, DFLNT, 3FL, LNFPII, and LNT (Supplementary Table S5). HMO profiles from secretor and non-secretor mothers were distinct (Figure 2B and Supplementary Table S5), in line with previous reports [28,29,30,31].

### 3.3. Influence of Human Milk Oligosaccharides on the Infant Oral Microbiome

Numerous small but statistically significant associations were identified between HMOs and infant oral microbiome features (Supplementary Tables S6 and S7).

**Figure 3:**
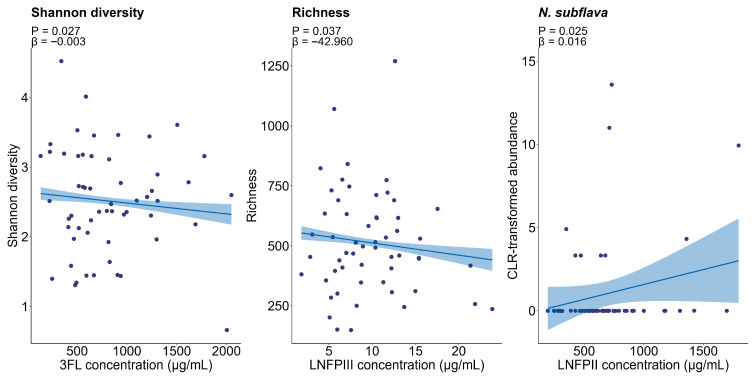
Associations between HMO concentration and the infant oral microbiome. Lines represent linear model fits, and shaded areas show 95% confidence intervals. HMO, human milk oligosaccharides.

We first assessed relationships between HMO concentrations and infant oral microbiome profiles. Concentrations of 3FL were negatively associated with infant oral Shannon diversity (estimate = −0.003, 95% CI: −0.004 to −0.001, *p* = 0.027), while LNFPIII concentration was negatively associated with richness (estimate = −42.960, 95% CI: −68.515 to −17.406, *p* = 0.037). The concentration of LNFPII was positively associated with *Neisseria subflava* (estimate = 0.016, 95% CI: 0.007 to 0.025, *p* = 0.025) (Figure 3).

**Figure 4:**
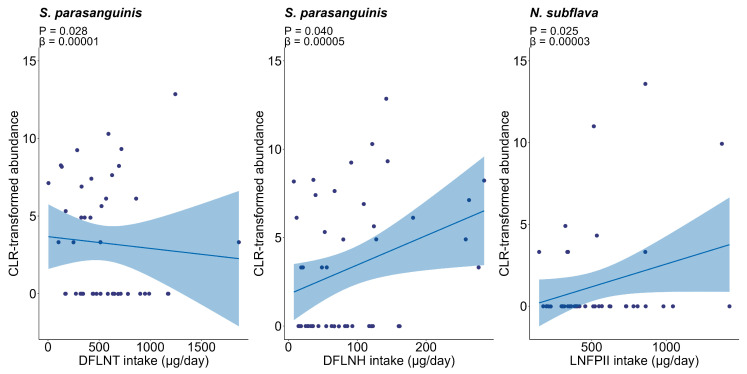
Associations between HMO intakes and infant oral taxa. Lines represent linear model fits, and shaded areas show 95% confidence intervals. HMO, human milk oligosaccharides.

We next assessed relationships between HMO intakes and the infant oral microbiome, identifying positive associations for both DFLNT and DFLNH with *Streptococcus parasanguinis* (estimate = 0.00001, 95% CI: 0.000008 to 0.00002, *p* = 0.028; and estimate = 0.00005, 95% CI: 0.00002 to 0.00009, *p* = 0.040, respectively), as well as a positive association between LNFPII and *N. subflava* (estimate = 0.00003, 95% CI: 0.000005 to 0.00003, *p* = 0.025) (Figure 4).

## 4. Discussion

Although HMOs have been extensively studied in relation to the human milk microbiome [32,33,34] and infant gut microbiome [7,8,9,35], their role in shaping the oral microbiota remains relatively underexplored. In fact, to our knowledge, there is only one other such study published to date, which examined a small subgroup of ten infants from the BLOSOM cohort [11]. The present study extends these findings by increasing the sample size and statistical rigour of the previous study. It is critical to understand the impact of milk bioactives, particularly prebiotic HMOs, on early oral microbiome development, as the establishment of beneficial oral bacteria acts to restrict the colonisation of potentially harmful species [36], shaping the early oral microbiome to support both oral and systemic health [37,38,39,40,41,42,43,44].

Our investigation into the relationships between HMO concentrations/intakes and the infant oral microbiome identified small, yet statistically significant associations with microbial composition among exclusively and predominantly breastfed infants. The oral microbiome in these infants was largely dominated by *S. mitis*, *G. haemolysans*, and *R. mucilaginosa*, consistent with previous findings [45,46,47,48,49]. Concentration of the fucosylated, non-sialylated oligosaccharide LNFPII was associated with higher levels of *N. subflava*. This species is commonly identified in the oral cavity and may contribute to early microbiome development [50]. The observed association suggests that LNFPII may facilitate colonisation or growth of specific commensal bacteria, possibly through selective nutrient availability or modulation of the local oral environment [5,51]. Although members of the *Neisseria* genus have a broad range of glycan utilisation genes [52,53], HMOs have previously been shown to inhibit the growth of pathogenic *Neisseria meningitidis* in vitro [54]. Nevertheless, interactions between commensal members of this genus and HMOs remain to be elucidated.

We also observed negative associations between concentrations of certain HMOs and measures of oral alpha diversity. Specifically, 3FL was negatively associated with Shannon diversity, while LNFPIII was negatively associated with richness. These findings suggest that HMOs may influence the infant oral microbiome, favouring the growth of specific taxa while limiting overall diversity. Reduced alpha diversity in early life has been observed in the oral microbiome and may reflect the selective enrichment of HMO-adapted microbial taxa, as has been described in the infant gut [6]. Both 3FL and LNFPIII contain an α1-3-linked fucose [55], which may provide a substrate for bacteria with α-1-3-fucosidase, allowing these taxa to dominate and shape overall community richness. HMOs may also modulate the infant oral microbiome indirectly through host immune mechanisms, influencing mucosal tolerance and microbial colonisation patterns. HMOs may act through multiple pathways, including direct antimicrobial activity, competitive nutrient utilisation, or modulation of microbial interactions [6,56]. However, the biological mechanisms underlying these associations remain unclear. Further longitudinal studies are needed to determine whether these HMO-microbiome associations have lasting implications for oral and systemic health.

The associations between DFLNT/DFLNH and *S. parasanguinis* reported here align with previous findings linking intakes of DFLac to *S. mitis* [11], and may reflect a broader affinity of oral streptococci for structurally related fucosylated oligosaccharides. The shared fucosylated motifs across DFLac, DFLNH, and DFLNT may provide common binding targets for streptococcal adhesins. *Streptococcus* spp. are known to possess fucosidase genes and have been associated with fucosylated HMOs in milk [57]. In vitro, *S. mitis* grows more rapidly in media supplemented with 2′FL or LNFPI [57], suggesting that fucosylated HMOs can serve as selective growth substrates. It is therefore plausible that DFLNT and DFLNH promote colonisation of *Streptococcus* spp. in the infant oral cavity via a similar mechanism. As early and dominant colonisers, *Streptococcus* spp. play a pivotal role in biofilm formation and in shaping microbial succession in the developing oral microbiome [58,59]. Our findings may therefore provide a mechanistic basis for the higher abundance of oral streptococci observed in exclusively breastfed compared with formula-fed infants [1,2], an effect potentially mediated by fucosylated HMOs.

A recent study involving a subset of 10 infants from the present cohort represents, to date, the only other investigation that has examined associations between daily HMO intakes and the composition of the infant oral microbiome [11]. The study identified multiple associations between HMO intakes and oral microbial taxa over the first four months of life, with patterns varying across time and differing by maternal secretor status. In contrast, our study examined a larger group of mothers and infants (*n* = 56) cross-sectionally. Our findings related to alpha diversity and *N. subflava* were not reported in Cheema et al.’s work, likely due to their stringent sub-sampling and low number of participants. However, in both studies, intakes of DFLNT were positively associated with members of the *Streptococcus* genus—*S. parasanguinis* in the case of the present study, and *S. mitis* in the Cheema et al. study. Together, these two studies offer complementary insights into the complex and time-dependent relationships between HMO intakes and the infant oral microbiome.

We have previously reported a dose-dependent relationship between human milk intake and the infant oral microbiome, with higher milk consumption associated with lower relative abundance of *S. parasanguinis* [13], an effect that may be partially mediated by HMOs. In the present analysis, we observed differential relationships between HMOs and the oral microbiome when assessing concentrations and intakes. This suggests that the influence of HMOs on the infant oral microbiome may be the result of both local and systemic effects. Supporting the potential relevance of HMO concentration, in vitro studies have shown that higher HMO concentrations more effectively inhibit biofilm formation and bacterial adhesion [60,61,62]. Our finding of divergent results for concentrations and intakes matches with previous work showing that concentrations and intakes of milk components can have different and often converse effects on infant outcomes [63]. These results emphasise the importance of measuring infant milk intake when considering the potential impact of milk components on infant health. While some statistically significant associations were modest in magnitude, even modest changes in early-life microbial composition may be biologically meaningful, particularly during critical periods of oral microbiome establishment. Human milk contains a complex mixture of nutrients and bioactive components, of which HMOs are just one group that collectively shape the infant oral microbiome. Therefore, it is not unexpected that any single component exerts only a modest influence on microbial composition. Nonetheless, these small effects may act cumulatively to support the development of a stable, health-promoting oral microbial community and could have downstream implications for long-term oral and systemic health, including immune development, although further longitudinal studies are needed to confirm their functional significance.

A key strength of this study lies in its assessment of both HMO concentrations and daily infant HMO intakes in relation to the infant oral microbiome. Additionally, the use of full-length 16S rRNA gene sequencing provides a greater taxonomic resolution and more precise microbial identification compared to short-read sequencing methods [64,65]. While our study identified associations between individual HMOs and the infant oral microbiome, it is important to emphasise that the observed effect sizes were extremely modest. Many of the associations did not remain statistically significant after correction for multiple comparisons and should therefore be interpreted cautiously. Furthermore, given the observational nature of our study, these findings cannot be considered causal. However, this study was limited by the availability of HMO data at a single time point and by a relatively small sample size (*n* = 56; *n* = 46 for HMO intake analyses), which may have reduced statistical power. Post hoc power calculations indicate that correlations of approximately r = 0.33–0.36 or larger could be reliably detected at α = 0.05 and 80% power, suggesting that smaller associations may not reach conventional levels of statistical significance and should be interpreted cautiously. Additionally, many of the HMOs measured were highly correlated (Supplementary Table S4), reflecting their shared biosynthetic pathways. This may limit the ability to disentangle the effects of individual HMOs on microbial outcomes and may affect the stability and direction of effect estimates, particularly for HMOs with high intercorrelations. In this study, we focused on individual HMOs and conducted correlation checks to interpret results carefully, but residual multicollinearity cannot be entirely excluded. Future studies involving larger and more diverse populations, as well as approaches such as grouped HMO families, principal component analysis, or penalised regression, are warranted to more fully characterise the impact of HMO concentrations and daily intakes on the development of the infant oral microbiome.

## 5. Conclusions

In summary, this study indicates that exposure to HMOs is associated with modest, measurable changes in the infant oral microbiome, which may contribute to the divergent oral microbiome profiles of breastfed and formula-fed infants [1,2,3], though the clinical and mechanistic implications of these effects remain uncertain. Larger, longitudinal studies are needed to elucidate the mechanisms driving these associations, which may have important implications for oral and systemic health across infancy and later life and inform potential interventions to improve breastfeeding success and oral health.

## Figures and Tables

**Table 1:** Participant characteristics.

Characteristic	Concentration Analysis (*n* = 56) Mean ± SD or *n* (%)	Intake Analysis (*n* = 46) Mean ± SD or *n* (%)
**Maternal age at delivery (years)**	32.3 ± 4.7	32.1 ± 5.0
**Maternal ethnicity**		
Caucasian	48 (86.0%)	41 (89.1%)
Other	8 (14.3%)	5 (11.0%)
**Delivery mode**		
Vaginal	38 (67.9%)	33 (71.7%)
Planned caesarean	13 (23.2%)	9 (19.6%)
Emergency caesarean	5 (8.9%)	4 (8.7%)
**Intrapartum antibiotic exposure**	25 (44.6%)	19 (41.3%)
**Maternal pre-pregnancy BMI (kg/m^2^)**		
Underweight	3 (5.4%)	2 (4.3%)
Normal	25 (44.6%)	22 (48.0%)
High	9 (16.1%)	8 (17.4%)
Obese	7 (12.5%)	6 (13.0%)
Missing	12 (21.4%)	8 (17.3%)
**Infant sex**		
Female	33 (59.0%)	27 (59.0%)
**Maternal secretor status**		
Secretor positive	47 (84.0%)	38 (83.0%)

## Data Availability

The original data presented in the study are openly available in NCBI Sequence Read Archive (SRA) under BioProject ID [PRJNA1186108] at http://www.ncbi.nlm.nih.gov/bioproject/1186108 (accessed on 6 August 2025).

## Supplementary Materials

The following supporting information can be downloaded at: https://www.mdpi.com/article/10.3390/nu17223622/s1, Table S1: Taxonomic assignments of OTUs based on SILVA and BLAST analyses; Table S2: Genera detected in negative extraction controls (EC, n = 8) and negative PCR controls (NTC, n = 8); Table S3: Normality of response variables assessed by Shapiro–Wilk test; Table S4: Variance inflation factors (VIFs) for predictors in models assessing associations between HMO concentrations and oral microbiome outcomes; Table S5: Comparison of human milk oligosaccharide concentrations between secretor and non-secretor mothers; Table S6: Outputs from linear model for associations between HMO concentrations and the infant oral microbiome at 2 months postpartum; Table S7: Outputs from linear model for associations between HMO intakes and the infant oral microbiome at 2 months postpartum.
