# Supplementary material for: Development of the Breastfed Infant Oral Microbiome Is Associated with Concentrations and Intakes of Human Milk Oligosaccharides
Source: Nutrients. 2025 Nov 20;17(22):3622. doi: 10.3390/nu17223622 (PMC12655218; doi:10.3390/nu17223622)
Supplement: Supplementary file 1 [file nutrients-17-03622-s001.zip › nutrients-3969298-supplementary.pdf]

Supplementary tables

Supplementry Table S1. Taxonomic assignments of OTUs based on SILVA and BLAST analyses.

OTU	SILVA genus assignment	BLAST species assignment	Sequnce ID	Identity accuracy	Coverage
Otu000001	*Streptococcus*	*Streptococcus mitis*	CP067992.1	98.58%	100%
Otu000002	*Gemella*	*Gemella haemolysans*	CP083637.1	99.53%	100%
Otu000003	*Rothia*	*Rothia mucilaginosa*	CP023510.1	99.66%	100%
Otu000004	*Streptococcus*	*Streptococcus salivarius* group 1	CP015283.1	97.63%	100%
Otu000005	*Veillonella*	*Veillonella* sp.	AY923122.1	97.86%	100%
Otu000006	*Neisseria*	*Neisseria subflava*	MF480369.1	99.52%	99%
Otu000007	*Streptococcus*	*Streptococcus oralis*	CP034442.1	97.84%	100%
Otu000008	*Haemophilus*	*Haemophilus haemolyticus*	CP031240.1	98.91%	100%
Otu000010	*Staphylococcus*	*Staphylococcus lugdunensis*	CP014023.2	100%	100%
Otu000011	*Haemophilus*	*Haemophilus parainfluenzae*	CP133470.1	98.51%	100%
Otu000013	*Streptococcus*	*Streptococcus parasanguinis*	CP134147.1	99.59%	100%
Otu000015	*Bifidobacterium*	*Bifidobacterium longum*	CP102541.1	99.86%	100%
Otu000016	*Lactobacillus*	*Lactobacillus gasseri*	CP054875.1	98.52%	99%
Otu000019	*Porphyromonas*	*Porphyromonas* sp.	DQ087194.1	99.52%	100%

Supplementary Table S2. Genera detected in negative extraction controls (EC, n = 8) and negative PCR controls (NTC, n = 8). Data are read numbers.

Genus	**EC1**	**EC2**	**EC3**	**EC4**	**EC5**	**EC6**	**EC7**	**EC8**	**NTC1**	**NTC2**	**NTC3**	**NTC4**	**NTC5**	**NTC6**	**NTC7**	**NTC8**
Actinomyces	2	2	0	0	3	1	1	0	1	1	0	0	1	1	0	0
Atopobium	2	1	0	1	0	0	0	0	0	1	2	0	0	0	0	0
Bergeyella	7	1	5	0	1	2	1	1	0	1	8	1	2	1	4	0
Gemella	37	0	1	0	0	0	1	1	1	0	1	2	2	0	3	1
Granulicatella	1	3	0	0	2	2	1	5	1	1	1	1	2	2	1	2
Haemophilus	3	0	0	0	0	0	0	0	0	0	0	0	0	0	0	0
Lactobacillus	1	1	1	0	1	1	0	0	0	1	3	0	1	1	0	1
Neisseria	8	2	5	2	5	4	1	2	0	4	2	1	9	2	2	3
Porphyromonas	9	0	0	0	0	0	0	0	0	1	1	0	2	1	0	0
Rothia	70	3	2	1	1	4	1	1	3	1	2	0	3	1	2	3
Unclassified *Saccharimonadales*	4	4	8	1	11	7	5	26	3	10	10	6	7	7	11	1
Sebaldella	1	0	0	0	0	1	0	0	0	0	0	0	0	0	0	0
Staphylococcus	3	0	3	0	6	5	1	0	2	4	1	2	3	2	5	0

Genus	**EC1**	**EC2**	**EC3**	**EC4**	**EC5**	**EC6**	**EC7**	**EC8**	**NTC1**	**NTC2**	**NTC3**	**NTC4**	**NTC5**	**NTC6**	**NTC7**	**NTC8**
Streptococcus	719	13	18	7	27	16	11	60	6	11	21	16	21	15	13	4
Unclassified *Actinobacteria*	2	2	0	0	0	3	1	0	0	0	3	1	1	1	1	0
Unclassified *Bacilli*	3	1	1	0	2	3	2	0	0	0	2	0	1	2	0	1
Unclassified bacteria	37	2	1	0	2	5	3	0	1	6	4	2	6	1	2	1
Unclassified *Carnobacteriaceae*	3	1	2	1	2	8	1	0	4	4	7	1	4	5	3	2
Unclassified *Lactobacillales*	7	4	2	1	12	18	6	0	0	1	3	4	9	8	11	3
Unclassified *Leptotrichiaceae*	1	1	0	0	3	0	0	3	1	0	2	0	2	0	1	0
Unclassified *Micrococcaceae*	6	0	0	0	0	0	0	0	0	0	2	0	0	0	0	0
Unclassified *Micrococcales*	25	5	6	2	2	9	2	4	3	4	8	6	8	4	4	2
Unclassified *Neisseriaceae*	1	0	4	2	3	4	61	9	0	0	0	0	0	1	1	2
Unclassified *Porphyromonadaceae*	1	3	1	0	4	1	3	1	1	2	0	1	7	2	4	0
Unclassified *Streptococcaceae*	33	1	4	0	2	0	0	0	0	1	1	0	0	0	2	0
Veillonella	549	8	9	2	6	7	6	18	3	8	4	11	8	8	5	2
Others*	1	7	6	9	51	57	95	33	6	13	4	9	41	47	27	15

*"Others" represents genera with ≤ 26 reads in negative controls.

Supplementary Table S3. Normality of response variables assessed by Shapiro–Wilk test.

**Response Variable**	**Shapiro–Wilk *p*-value**	**Normality**
Shannon diversity	0.1946	Yes
Richness	0.6882	Yes
Streptococcus mitis	0.0179	No
Gemella haemolysans	0.7040	Yes
Rothia mucilaginosa	0.6011	Yes
Streptococcus salivarius group 1	0.1052	Yes
Veillonella sp.	0.5389	Yes
Neisseria subflava	0.2784	Yes
Streptococcus oralis	0.4067	Yes
Haemophilus haemolyticus	0.1245	Yes
Veillonella nakazawae	0.5227	Yes
Staphylococcus lugdunensis	0.0070	No
Haemophilus parainfluenzae	0.5103	Yes
Streptococcus parasanguinis	0.6155	Yes
Bifidobacterium longum	0.0130	No
Lactobacillus gasseri	0.9341	Yes
Porphyromonas sp.	0.0005	No

Supplementary Table S4. Variance inflation factors (VIFs) for predictors in models assessing associations between HMO concentrations and oral microbiome outcomes.

Response	Variable	VIF
Shannon diversity	2’FL	13.6
Shannon diversity	3FL	19.779
Shannon diversity	DFLac	7.868
Shannon diversity	3’SL	2.173
Shannon diversity	6’SL	8.989
Shannon diversity	LNT	19.974
Shannon diversity	LNnT	11.62
Shannon diversity	LNFPI	13.028
Shannon diversity	LNFPII	39.783
Shannon diversity	LNFPIII	4.399
Shannon diversity	LSTb	10.286
Shannon diversity	LSTc	9.744
Shannon diversity	DFLNT	12.214
Shannon diversity	LNH	4.649
Shannon diversity	DSLNT	6.645
Shannon diversity	FLNH	10.525
Shannon diversity	DFLNH	4.931
Shannon diversity	FDSLNH	19.056
Shannon diversity	DSLNH	10.923
Richness	2’FL	13.6
Richness	3FL	19.779
Richness	DFLac	7.868
Richness	3’SL	2.173
Richness	6’SL	8.989
Richness	LNT	19.974
Richness	LNnT	11.62
Richness	LNFPI	13.028
Richness	LNFPII	39.783
Richness	LNFPIII	4.399
Richness	LSTb	10.286
Richness	LSTc	9.744
Richness	DFLNT	12.214
Richness	LNH	4.649
Richness	DSLNT	6.645
Richness	FLNH	10.525
Richness	DFLNH	4.931
Richness	FDSLNH	19.056
Richness	DSLNH	10.923
Streptococcus mitis	2’FL	13.6

Streptococcus mitis	3FL	19.779
Streptococcus mitis	DFLac	7.868
Streptococcus mitis	3’SL	2.173
Streptococcus mitis	6’SL	8.989
Streptococcus mitis	LNT	19.974
Streptococcus mitis	LNnT	11.62
Streptococcus mitis	LNFPI	13.028
Streptococcus mitis	LNFPII	39.783
Streptococcus mitis	LNFPIII	4.399
Streptococcus mitis	LSTb	10.286
Streptococcus mitis	LSTc	9.744
Streptococcus mitis	DFLNT	12.214
Streptococcus mitis	LNH	4.649
Streptococcus mitis	DSLNT	6.645
Streptococcus mitis	FLNH	10.525
Streptococcus mitis	DFLNH	4.931
Streptococcus mitis	FDSLNH	19.056
Streptococcus mitis	DSLNH	10.923
Gemella haemolysans	2’FL	13.6
Gemella haemolysans	3FL	19.779
Gemella haemolysans	DFLac	7.868
Gemella haemolysans	3’SL	2.173
Gemella haemolysans	6’SL	8.989
Gemella haemolysans	LNT	19.974
Gemella haemolysans	LNnT	11.62
Gemella haemolysans	LNFPI	13.028
Gemella haemolysans	LNFPII	39.783
Gemella haemolysans	LNFPIII	4.399
Gemella haemolysans	LSTb	10.286
Gemella haemolysans	LSTc	9.744
Gemella haemolysans	DFLNT	12.214
Gemella haemolysans	LNH	4.649
Gemella haemolysans	DSLNT	6.645
Gemella haemolysans	FLNH	10.525
Gemella haemolysans	DFLNH	4.931
Gemella haemolysans	FDSLNH	19.056
Gemella haemolysans	DSLNH	10.923
Streptococcus salivarius group 1	2’FL	13.6
Streptococcus salivarius group 1	3FL	19.779
Streptococcus salivarius group 1	DFLac	7.868
Streptococcus salivarius group 1	3’SL	2.173
Streptococcus salivarius group 1	6’SL	8.989

Streptococcus salivarius group 1	LNT	19.974
Streptococcus salivarius group 1	LNnT	11.62
Streptococcus salivarius group 1	LNFPI	13.028
Streptococcus salivarius group 1	LNFPII	39.783
Streptococcus salivarius group 1	LNFPIII	4.399
Streptococcus salivarius group 1	LSTb	10.286
Streptococcus salivarius group 1	LSTc	9.744
Streptococcus salivarius group 1	DFLNT	12.214
Streptococcus salivarius group 1	LNH	4.649
Streptococcus salivarius group 1	DSLNT	6.645
Streptococcus salivarius group 1	FLNH	10.525
Streptococcus salivarius group 1	DFLNH	4.931
Streptococcus salivarius group 1	FDSLNH	19.056
Streptococcus salivarius group 1	DSLNH	10.923
Rothia mucilaginosa	2’FL	13.6
Rothia mucilaginosa	3FL	19.779
Rothia mucilaginosa	DFLac	7.868
Rothia mucilaginosa	3’SL	2.173
Rothia mucilaginosa	6’SL	8.989
Rothia mucilaginosa	LNT	19.974
Rothia mucilaginosa	LNnT	11.62
Rothia mucilaginosa	LNFPI	13.028
Rothia mucilaginosa	LNFPII	39.783
Rothia mucilaginosa	LNFPIII	4.399
Rothia mucilaginosa	LSTb	10.286
Rothia mucilaginosa	LSTc	9.744
Rothia mucilaginosa	DFLNT	12.214
Rothia mucilaginosa	LNH	4.649
Rothia mucilaginosa	DSLNT	6.645
Rothia mucilaginosa	FLNH	10.525
Rothia mucilaginosa	DFLNH	4.931
Rothia mucilaginosa	FDSLNH	19.056
Rothia mucilaginosa	DSLNH	10.923
Veillonella sp.	2’FL	13.6
Veillonella sp.	3FL	19.779
Veillonella sp.	DFLac	7.868
Veillonella sp.	3’SL	2.173
Veillonella sp.	6’SL	8.989
Veillonella sp.	LNT	19.974
Veillonella sp.	LNnT	11.62
Veillonella sp.	LNFPI	13.028
Veillonella sp.	LNFPII	39.783

Veillonella sp.	LNFPIII	4.399
Veillonella sp.	LSTb	10.286
Veillonella sp.	LSTc	9.744
Veillonella sp.	DFLNT	12.214
Veillonella sp.	LNH	4.649
Veillonella sp.	DSLNT	6.645
Veillonella sp.	FLNH	10.525
Veillonella sp.	DFLNH	4.931
Veillonella sp.	FDSLNH	19.056
Veillonella sp.	DSLNH	10.923
Veillonella nakazawae	2'FL	13.6
Veillonella nakazawae	3FL	19.779
Veillonella nakazawae	DFLac	7.868
Veillonella nakazawae	3'SL	2.173
Veillonella nakazawae	6'SL	8.989
Veillonella nakazawae	LNT	19.974
Veillonella nakazawae	LNnT	11.62
Veillonella nakazawae	LNFPI	13.028
Veillonella nakazawae	LNFPII	39.783
Veillonella nakazawae	LNFPIII	4.399
Veillonella nakazawae	LSTb	10.286
Veillonella nakazawae	LSTc	9.744
Veillonella nakazawae	DFLNT	12.214
Veillonella nakazawae	LNH	4.649
Veillonella nakazawae	DSLNT	6.645
Veillonella nakazawae	FLNH	10.525
Veillonella nakazawae	DFLNH	4.931
Veillonella nakazawae	FDSLNH	19.056
Veillonella nakazawae	DSLNH	10.923
Lactobacillus gasseri	2'FL	13.6
Lactobacillus gasseri	3FL	19.779
Lactobacillus gasseri	DFLac	7.868
Lactobacillus gasseri	3'SL	2.173
Lactobacillus gasseri	6'SL	8.989
Lactobacillus gasseri	LNT	19.974
Lactobacillus gasseri	LNnT	11.62
Lactobacillus gasseri	LNFPI	13.028
Lactobacillus gasseri	LNFPII	39.783
Lactobacillus gasseri	LNFPIII	4.399
Lactobacillus gasseri	LSTb	10.286
Lactobacillus gasseri	LSTc	9.744
Lactobacillus gasseri	DFLNT	12.214

Lactobacillus gasseri	LNH	4.649
Lactobacillus gasseri	DSLNT	6.645
Lactobacillus gasseri	FLNH	10.525
Lactobacillus gasseri	DFLNH	4.931
Lactobacillus gasseri	FDSLNH	19.056
Lactobacillus gasseri	DSLNH	10.923
Haemophilus haemolyticus	2’FL	13.6
Haemophilus haemolyticus	3FL	19.779
Haemophilus haemolyticus	DFLac	7.868
Haemophilus haemolyticus	3’SL	2.173
Haemophilus haemolyticus	6’SL	8.989
Haemophilus haemolyticus	LNT	19.974
Haemophilus haemolyticus	LNnT	11.62
Haemophilus haemolyticus	LNFPI	13.028
Haemophilus haemolyticus	LNFPII	39.783
Haemophilus haemolyticus	LNFPIII	4.399
Haemophilus haemolyticus	LSTb	10.286
Haemophilus haemolyticus	LSTc	9.744
Haemophilus haemolyticus	DFLNT	12.214
Haemophilus haemolyticus	LNH	4.649
Haemophilus haemolyticus	DSLNT	6.645
Haemophilus haemolyticus	FLNH	10.525
Haemophilus haemolyticus	DFLNH	4.931
Haemophilus haemolyticus	FDSLNH	19.056
Haemophilus haemolyticus	DSLNH	10.923
Haemophilus parainfluenzae	2’FL	13.6
Haemophilus parainfluenzae	3FL	19.779
Haemophilus parainfluenzae	DFLac	7.868
Haemophilus parainfluenzae	3’SL	2.173
Haemophilus parainfluenzae	6’SL	8.989
Haemophilus parainfluenzae	LNT	19.974
Haemophilus parainfluenzae	LNnT	11.62
Haemophilus parainfluenzae	LNFPI	13.028
Haemophilus parainfluenzae	LNFPII	39.783
Haemophilus parainfluenzae	LNFPIII	4.399
Haemophilus parainfluenzae	LSTb	10.286
Haemophilus parainfluenzae	LSTc	9.744
Haemophilus parainfluenzae	DFLNT	12.214
Haemophilus parainfluenzae	LNH	4.649
Haemophilus parainfluenzae	DSLNT	6.645
Haemophilus parainfluenzae	FLNH	10.525
Haemophilus parainfluenzae	DFLNH	4.931

Haemophilus parainfluenzae	FDSLNH	19.056
Haemophilus parainfluenzae	DSLNH	10.923
Streptococcus oralis	2’FL	13.6
Streptococcus oralis	3FL	19.779
Streptococcus oralis	DFLac	7.868
Streptococcus oralis	3’SL	2.173
Streptococcus oralis	6’SL	8.989
Streptococcus oralis	LNT	19.974
Streptococcus oralis	LNnT	11.62
Streptococcus oralis	LNFPI	13.028
Streptococcus oralis	LNFPII	39.783
Streptococcus oralis	LNFPIII	4.399
Streptococcus oralis	LSTb	10.286
Streptococcus oralis	LSTc	9.744
Streptococcus oralis	DFLNT	12.214
Streptococcus oralis	LNH	4.649
Streptococcus oralis	DSLNT	6.645
Streptococcus oralis	FLNH	10.525
Streptococcus oralis	DFLNH	4.931
Streptococcus oralis	FDSLNH	19.056
Streptococcus oralis	DSLNH	10.923
Bifidobacterium longum	2’FL	13.6
Bifidobacterium longum	3FL	19.779
Bifidobacterium longum	DFLac	7.868
Bifidobacterium longum	3’SL	2.173
Bifidobacterium longum	6’SL	8.989
Bifidobacterium longum	LNT	19.974
Bifidobacterium longum	LNnT	11.62
Bifidobacterium longum	LNFPI	13.028
Bifidobacterium longum	LNFPII	39.783
Bifidobacterium longum	LNFPIII	4.399
Bifidobacterium longum	LSTb	10.286
Bifidobacterium longum	LSTc	9.744
Bifidobacterium longum	DFLNT	12.214
Bifidobacterium longum	LNH	4.649
Bifidobacterium longum	DSLNT	6.645
Bifidobacterium longum	FLNH	10.525
Bifidobacterium longum	DFLNH	4.931
Bifidobacterium longum	FDSLNH	19.056
Bifidobacterium longum	DSLNH	10.923
Staphylococcus lugdunensis	2’FL	13.6
Staphylococcus lugdunensis	3FL	19.779

Staphylococcus lugdunensis	DFLac	7.868
Staphylococcus lugdunensis	3'SL	2.173
Staphylococcus lugdunensis	6'SL	8.989
Staphylococcus lugdunensis	LNT	19.974
Staphylococcus lugdunensis	LNnT	11.62
Staphylococcus lugdunensis	LNFPI	13.028
Staphylococcus lugdunensis	LNFPII	39.783
Staphylococcus lugdunensis	LNFPIII	4.399
Staphylococcus lugdunensis	LSTb	10.286
Staphylococcus lugdunensis	LSTc	9.744
Staphylococcus lugdunensis	DFLNT	12.214
Staphylococcus lugdunensis	LNH	4.649
Staphylococcus lugdunensis	DSLNT	6.645
Staphylococcus lugdunensis	FLNH	10.525
Staphylococcus lugdunensis	DFLNH	4.931
Staphylococcus lugdunensis	FDSLNH	19.056
Staphylococcus lugdunensis	DSLNH	10.923
Streptococcus parasanguinis	2'FL	13.6
Streptococcus parasanguinis	3FL	19.779
Streptococcus parasanguinis	DFLac	7.868
Streptococcus parasanguinis	3'SL	2.173
Streptococcus parasanguinis	6'SL	8.989
Streptococcus parasanguinis	LNT	19.974
Streptococcus parasanguinis	LNnT	11.62
Streptococcus parasanguinis	LNFPI	13.028
Streptococcus parasanguinis	LNFPII	39.783
Streptococcus parasanguinis	LNFPIII	4.399
Streptococcus parasanguinis	LSTb	10.286
Streptococcus parasanguinis	LSTc	9.744
Streptococcus parasanguinis	DFLNT	12.214
Streptococcus parasanguinis	LNH	4.649
Streptococcus parasanguinis	DSLNT	6.645
Streptococcus parasanguinis	FLNH	10.525
Streptococcus parasanguinis	DFLNH	4.931
Streptococcus parasanguinis	FDSLNH	19.056
Streptococcus parasanguinis	DSLNH	10.923
Neisseria subflava	2'FL	13.6
Neisseria subflava	3FL	19.779
Neisseria subflava	DFLac	7.868
Neisseria subflava	3'SL	2.173
Neisseria subflava	6'SL	8.989
Neisseria subflava	LNT	19.974

Neisseria subflava	LNnT	11.62
Neisseria subflava	LNFPI	13.028
Neisseria subflava	LNFPII	39.783
Neisseria subflava	LNFPIII	4.399
Neisseria subflava	LSTb	10.286
Neisseria subflava	LSTc	9.744
Neisseria subflava	DFLNT	12.214
Neisseria subflava	LNH	4.649
Neisseria subflava	DSLNT	6.645
Neisseria subflava	FLNH	10.525
Neisseria subflava	DFLNH	4.931
Neisseria subflava	FDSLNH	19.056
Neisseria subflava	DSLNH	10.923
Porphyromonas sp.	2'FL	13.6
Porphyromonas sp.	3FL	19.779
Porphyromonas sp.	DFLac	7.868
Porphyromonas sp.	3'SL	2.173
Porphyromonas sp.	6'SL	8.989
Porphyromonas sp.	LNT	19.974
Porphyromonas sp.	LNnT	11.62
Porphyromonas sp.	LNFPI	13.028
Porphyromonas sp.	LNFPII	39.783
Porphyromonas sp.	LNFPIII	4.399
Porphyromonas sp.	LSTb	10.286
Porphyromonas sp.	LSTc	9.744
Porphyromonas sp.	DFLNT	12.214
Porphyromonas sp.	LNH	4.649
Porphyromonas sp.	DSLNT	6.645
Porphyromonas sp.	FLNH	10.525
Porphyromonas sp.	DFLNH	4.931
Porphyromonas sp.	FDSLNH	19.056
Porphyromonas sp.	DSLNH	10.923

Supplementary Table S5. Comparison of human milk oligosaccharide concentrations between secretor and non-secretor mothers. Data are median ± IQR. Bold text indicated significant *P*-values.

HMO	Secretors (median ± IQR)	Non-secretors (median ± IQR)	*P*-value	BH corrected *P*-value
2’FL	1783.8 ± 955.4	1.7 ± 1.8	0.000	**0.000**
DFLac	204.7 ± 103.2	3.9 ± 2.1	0.000	**0.000**
3FL	605.1 ± 372.1	1620.1 ± 480.0	0.000	**0.000**
LNFPII	580.6 ± 249.1	1303.2 ± 506.6	0.000	**0.000**
FDSLNH	160.4 ± 161.7	655.06± 331.7	0.000	**0.000**
DFLNT	773.3 ± 380.4	261.6 ± 170.6	0.000	**0.000**
LNFPI	389.8 ± 491.6	119.5 ± 64.8	0.000	**0.000**
LNnT	98.7 ± 68.9	41.5 ± 15.9	0.000	**0.000**
LSTc	73.0 ± 51.7	39.3 ± 8.5	0.001	**0.001**
LNT	562.4 ± 339.5	847.3 ± 585.0	0.051	0.097
LSTb	56.4 ± 36.4	76.7 ± 24.4	0.063	0.109
LNH	57.3 ± 32.1	47.1 ± 19.3	0.090	0.143
FLNH	189.74± 106.8	253.0 ± 169.2	0.199	0.291
DFLNH	78.0 ± 89.6	102.5 ± 62.7	0.224	0.304
3’SL	92.12± 45.6	71.4 ± 58.1	0.441	0.559
DSLNH	130.01± 80.6	121.8 ± 76.9	0.742	0.854
LNFPIII	8.7 ± 6.3	8.9 ± 4.8	0.776	0.854
DSLNT	111.5 ± 69.2	117.9 ± 31.8	0.809	0.854
6’SL	153.9 ± 89.6	151.5 ± 59.7	0.930	0.930

2'-fucosyllactose (2'FL), 3-fucosyllactose (3FL), difucosyllactose (DFLac), 3'-sialyllactose (3'SL), 6'-sialyllactose (6'SL), difucosyl-N-hexosyl (DFLNH), difucosyl-N-acetyl (DFLNT), disialyl-N-hexosyl (DSLNH), disialyl-N-acetyl (DSLNT), fucosyl-disialyl-N-hexosyl (FDSLNH), fucosyllacto-N-hexaose (FLNH), lacto-N-fucopentaose I (LNFP I), lacto-N-fucopentaose II (LNFP II), lacto-N-fucopentaose III (LNFP III), lacto-N-hexaose (LNH), lacto-N-neotetraose (LNnT), lacto-N-tetraose (LNT), lacto-N-neotriose (LSTc), lacto-N-neotriose (LSTb), Median concentrations (µg/mL) and interquartile ranges (IQR) of individual HMOs in secretor (S) and non-secretor (NS) groups. P-values were calculated using the Wilcoxon rank-sum test.

Supplementary Table S6. Outputs from linear model for associations between HMO concentrations and the infant oral microbiome at 2 months postpartum. Bold text indicated significant *P*-values.

Response variable	Explanatory variable	Estimate	Standard Error	*P*-value	BH corrected *P*-value	Lower CI	Upper CI
Shannon diversity	Secretor status: Secretor	-1.911	0.674	0.008	0.078	-3.232	-0.590
Shannon diversity	2'FL	0.000	0.000	0.286	0.535	-0.001	0.000
Shannon diversity	3FL	-0.003	0.001	0.002	**0.027**	-0.004	-0.001
Shannon diversity	DFLac	0.002	0.001	0.103	0.358	0.000	0.005
Shannon diversity	3'SL	0.003	0.003	0.350	0.577	-0.003	0.008
Shannon diversity	6'SL	0.003	0.003	0.276	0.535	-0.002	0.009
Shannon diversity	LNT	-0.001	0.001	0.442	0.597	-0.004	0.002
Shannon diversity	LNnT	0.003	0.005	0.491	0.597	-0.006	0.013
Shannon diversity	LNFPI	0.000	0.001	0.988	0.988	-0.002	0.002
Shannon diversity	LNFPII	0.001	0.001	0.490	0.597	-0.002	0.004
Shannon diversity	LNFPIII	-0.097	0.046	0.046	0.276	-0.187	-0.006
Shannon diversity	LSTb	0.007	0.008	0.433	0.597	-0.010	0.023
Shannon diversity	LSTc	-0.008	0.008	0.309	0.540	-0.024	0.007
Shannon diversity	DFLNT	0.000	0.001	0.766	0.858	-0.002	0.001
Shannon diversity	LNH	0.010	0.007	0.153	0.358	-0.003	0.023
Shannon diversity	DSLNT	0.003	0.005	0.620	0.723	-0.008	0.013
Shannon diversity	FLNH	-0.005	0.003	0.151	0.358	-0.010	0.001
Shannon diversity	DFLNH	0.000	0.002	0.816	0.879	-0.005	0.004
Shannon diversity	FDSLNH	0.000	0.002	0.974	0.988	-0.003	0.003
Shannon diversity	DSLNH	0.009	0.005	0.065	0.276	0.000	0.018
Shannon diversity	Delivery mode: emergency caesarean	0.536	0.460	0.254	0.535	-0.365	1.437
Shannon diversity	Delivery mode: planned caesarean	-0.430	0.276	0.130	0.358	-0.970	0.110
Shannon diversity	First week pacifier: yes	0.633	0.335	0.069	0.276	-0.023	1.290
Shannon diversity	Siblings: yes	0.232	0.311	0.461	0.597	-0.378	0.842
Shannon diversity	Pre-pregnancy BMI: high and obese	-0.406	0.270	0.143	0.358	-0.936	0.123
Richness	Secretor status: Secretor	-529.672	190.174	0.009	0.066	-902.412	-156.931

Richness	2’FL	-0.046	0.094	0.624	0.760	-0.230	0.137
Richness	3FL	-0.699	0.231	0.005	0.050	-1.152	-0.245
Richness	DFLac	0.718	0.381	0.070	0.313	-0.029	1.465
Richness	3’SL	1.105	0.805	0.181	0.422	-0.473	2.683
Richness	6’SL	1.017	0.815	0.222	0.478	-0.579	2.614
Richness	LNT	-0.017	0.368	0.964	0.964	-0.738	0.704
Richness	LNnT	0.749	1.393	0.595	0.757	-1.981	3.478
Richness	LNFPI	0.071	0.273	0.798	0.894	-0.464	0.605
Richness	LNFPII	0.143	0.398	0.722	0.843	-0.638	0.924
Richness	LNFPIII	-42.961	13.038	0.003	**0.037**	-68.515	-17.406
Richness	LSTb	0.108	2.395	0.964	0.964	-4.587	4.803
Richness	LSTc	-2.201	2.278	0.342	0.532	-6.666	2.263
Richness	DFLNT	-0.245	0.220	0.275	0.481	-0.676	0.186
Richness	LNH	3.184	1.912	0.107	0.333	-0.563	6.931
Richness	DSLNT	2.721	1.502	0.081	0.313	-0.223	5.664
Richness	FLNH	-1.516	0.862	0.090	0.313	-3.206	0.173
Richness	DFLNH	-0.360	0.599	0.553	0.757	-1.534	0.815
Richness	FDSLNH	0.086	0.434	0.844	0.909	-0.764	0.937
Richness	DSLNH	1.547	1.308	0.247	0.481	-1.017	4.111
Richness	Delivery mode: emergency caesarean	105.712	129.692	0.422	0.622	-148.485	359.909
Richness	Delivery mode: planned caesarean	-153.969	77.791	0.058	0.313	-306.439	-1.498
Richness	First week pacifier: yes	136.359	94.496	0.160	0.408	-48.852	321.571
Richness	Siblings: yes	94.271	87.798	0.292	0.481	-77.813	266.354
Richness	Pre-pregnancy BMI: high and obese	-85.561	76.168	0.271	0.481	-234.851	63.728
Streptococcus mitis	Secretor status: Secretor	3.661	2.485	0.152	0.894	-1.210	8.532
Streptococcus mitis	2’FL	0.001	0.001	0.520	0.968	-0.002	0.003
Streptococcus mitis	3FL	0.002	0.003	0.591	0.968	-0.004	0.008
Streptococcus mitis	DFLac	-0.004	0.005	0.384	0.968	-0.014	0.005
Streptococcus mitis	3’SL	-0.005	0.011	0.654	0.968	-0.025	0.016

Streptococcus mitis	6'SL	-0.015	0.011	0.160	0.894	-0.036	0.005
Streptococcus mitis	LNT	0.001	0.005	0.888	0.968	-0.009	0.010
Streptococcus mitis	LNnT	-0.033	0.018	0.083	0.894	-0.068	0.003
Streptococcus mitis	LNFPI	-0.001	0.004	0.822	0.968	-0.008	0.006
Streptococcus mitis	LNFPII	0.006	0.005	0.284	0.968	-0.005	0.016
Streptococcus mitis	LNFPIII	0.050	0.170	0.773	0.968	-0.284	0.384
Streptococcus mitis	LSTb	-0.017	0.031	0.590	0.968	-0.078	0.044
Streptococcus mitis	LSTc	0.036	0.030	0.238	0.953	-0.022	0.094
Streptococcus mitis	DFLNT	0.000	0.003	0.968	0.968	-0.006	0.006
Streptococcus mitis	LNH	0.002	0.025	0.930	0.968	-0.047	0.051
Streptococcus mitis	DSLNT	-0.003	0.020	0.897	0.968	-0.041	0.036
Streptococcus mitis	FLNH	0.012	0.011	0.314	0.968	-0.011	0.034
Streptococcus mitis	DFLNH	-0.003	0.008	0.730	0.968	-0.018	0.013
Streptococcus mitis	FDSLNH	-0.007	0.006	0.232	0.953	-0.018	0.004
Streptococcus mitis	DSLNH	-0.003	0.017	0.861	0.968	-0.037	0.030
Streptococcus mitis	Delivery mode: emergency caesarean	0.692	1.695	0.686	0.968	-2.630	4.014
Streptococcus mitis	Delivery mode: planned caesarean	1.474	1.017	0.158	0.894	-0.518	3.466
Streptococcus mitis	First week pacifier: yes	-0.728	1.235	0.560	0.968	-3.148	1.692
Streptococcus mitis	Siblings: yes	-0.065	1.147	0.956	0.968	-2.313	2.184
Streptococcus mitis	Pre-pregnancy BMI: high and obese	-0.585	0.995	0.562	0.968	-2.536	1.366
Gemella haemolysans	Secretor status: Secretor	0.348	3.188	0.914	0.951	-5.900	6.595
Gemella haemolysans	2'FL	-0.001	0.002	0.649	0.951	-0.004	0.002
Gemella haemolysans	3FL	-0.008	0.004	0.060	0.380	-0.015	0.000
Gemella haemolysans	DFLac	0.003	0.006	0.695	0.951	-0.010	0.015
Gemella haemolysans	3'SL	-0.008	0.013	0.542	0.951	-0.035	0.018
Gemella haemolysans	6'SL	0.005	0.014	0.701	0.951	-0.021	0.032
Gemella haemolysans	LNT	-0.002	0.006	0.690	0.951	-0.015	0.010
Gemella haemolysans	LNnT	-0.015	0.023	0.536	0.951	-0.060	0.031
Gemella haemolysans	LNFPI	0.001	0.005	0.904	0.951	-0.008	0.010

Gemella haemolysans	LNFPII	0.019	0.007	0.010	0.135	0.005	0.032
Gemella haemolysans	LNFPIII	-0.024	0.219	0.914	0.951	-0.452	0.405
Gemella haemolysans	LSTb	-0.046	0.040	0.257	0.901	-0.125	0.032
Gemella haemolysans	LSTc	0.001	0.038	0.989	0.989	-0.074	0.075
Gemella haemolysans	DFLNT	-0.002	0.004	0.560	0.951	-0.009	0.005
Gemella haemolysans	LNH	0.011	0.032	0.733	0.951	-0.052	0.074
Gemella haemolysans	DSLNT	-0.030	0.025	0.250	0.901	-0.079	0.020
Gemella haemolysans	FLNH	0.006	0.014	0.686	0.951	-0.022	0.034
Gemella haemolysans	DFLNH	-0.017	0.010	0.100	0.468	-0.037	0.003
Gemella haemolysans	FDSLNH	-0.016	0.007	0.032	0.295	-0.031	-0.002
Gemella haemolysans	DSLNH	-0.002	0.022	0.917	0.951	-0.045	0.041
Gemella haemolysans	Delivery mode: emergency caesarean	4.128	2.174	0.068	0.380	-0.133	8.388
Gemella haemolysans	Delivery mode: planned caesarean	0.481	1.304	0.715	0.951	-2.075	3.036
Gemella haemolysans	First week pacifier: yes	0.777	1.584	0.627	0.951	-2.327	3.882
Gemella haemolysans	Siblings: yes	1.553	1.472	0.300	0.935	-1.332	4.437
Gemella haemolysans	Pre-pregnancy BMI: high and obese	0.357	1.277	0.782	0.951	-2.146	2.859
Rothia mucilaginosa	Secretor status: Secretor	0.216	3.671	0.953	0.953	-6.979	7.411
Rothia mucilaginosa	2’FL	0.002	0.002	0.380	0.861	-0.002	0.005
Rothia mucilaginosa	3FL	-0.001	0.004	0.831	0.924	-0.010	0.008
Rothia mucilaginosa	DFLac	-0.002	0.007	0.744	0.906	-0.017	0.012
Rothia mucilaginosa	3’SL	-0.021	0.016	0.190	0.731	-0.051	0.010
Rothia mucilaginosa	6’SL	-0.010	0.016	0.548	0.861	-0.040	0.021
Rothia mucilaginosa	LNT	-0.011	0.007	0.122	0.671	-0.025	0.003
Rothia mucilaginosa	LNnT	-0.005	0.027	0.848	0.924	-0.058	0.047
Rothia mucilaginosa	LNFPI	-0.010	0.005	0.063	0.592	-0.020	0.000
Rothia mucilaginosa	LNFPII	-0.004	0.008	0.619	0.861	-0.019	0.011
Rothia mucilaginosa	LNFPIII	0.379	0.252	0.144	0.671	-0.115	0.872
Rothia mucilaginosa	LSTb	0.130	0.046	0.009	0.255	0.039	0.220
Rothia mucilaginosa	LSTc	0.015	0.044	0.737	0.906	-0.071	0.101

Rothia mucilaginosa	DFLNT	0.003	0.004	0.557	0.861	-0.006	0.011
Rothia mucilaginosa	LNH	-0.028	0.037	0.453	0.861	-0.100	0.044
Rothia mucilaginosa	DSLNT	-0.020	0.029	0.487	0.861	-0.077	0.036
Rothia mucilaginosa	FLNH	0.010	0.017	0.559	0.861	-0.023	0.042
Rothia mucilaginosa	DFLNH	0.010	0.012	0.404	0.861	-0.013	0.032
Rothia mucilaginosa	FDSLNH	0.011	0.008	0.209	0.731	-0.006	0.027
Rothia mucilaginosa	DSLNH	0.005	0.025	0.858	0.924	-0.045	0.054
Rothia mucilaginosa	Delivery mode: emergency caesarean	-1.163	2.503	0.646	0.861	-6.070	3.744
Rothia mucilaginosa	Delivery mode: planned caesarean	2.293	1.502	0.138	0.671	-0.650	5.236
Rothia mucilaginosa	First week pacifier: yes	0.174	1.824	0.925	0.953	-3.401	3.749
Rothia mucilaginosa	Siblings: yes	1.001	1.695	0.559	0.861	-2.320	4.323
Rothia mucilaginosa	Pre-pregnancy BMI: high and obese	1.701	1.470	0.257	0.800	-1.181	4.582
Streptococcus salivarius group 1	Secretor status: Secretor	-3.375	4.694	0.478	0.970	-12.576	5.826
Streptococcus salivarius group 1	2'FL	0.001	0.002	0.612	0.970	-0.003	0.006
Streptococcus salivarius group 1	3FL	0.005	0.006	0.354	0.970	-0.006	0.017
Streptococcus salivarius group 1	DFLac	0.001	0.009	0.947	0.970	-0.018	0.019
Streptococcus salivarius group 1	3'SL	-0.041	0.020	0.046	0.970	-0.080	-0.003
Streptococcus salivarius group 1	6'SL	-0.001	0.020	0.970	0.970	-0.040	0.039
Streptococcus salivarius group 1	LNT	0.002	0.009	0.865	0.970	-0.016	0.019
Streptococcus salivarius group 1	LNnT	0.052	0.034	0.138	0.970	-0.015	0.120
Streptococcus salivarius group 1	LNFPI	0.000	0.007	0.941	0.970	-0.014	0.013
Streptococcus salivarius group 1	LNFPII	-0.011	0.010	0.271	0.970	-0.030	0.008
Streptococcus salivarius group 1	LNFPIII	-0.111	0.322	0.734	0.970	-0.741	0.520
Streptococcus salivarius group 1	LSTb	0.046	0.059	0.440	0.970	-0.070	0.162
Streptococcus salivarius group 1	LSTc	0.019	0.056	0.737	0.970	-0.091	0.129
Streptococcus salivarius group 1	DFLNT	0.000	0.005	0.955	0.970	-0.011	0.010

Streptococcus salivarius group 1	LNH	-0.087	0.047	0.076	0.970	-0.179	0.005
Streptococcus salivarius group 1	DSLNT	-0.009	0.037	0.815	0.970	-0.081	0.064
Streptococcus salivarius group 1	FLNH	0.014	0.021	0.516	0.970	-0.028	0.056
Streptococcus salivarius group 1	DFLNH	0.010	0.015	0.517	0.970	-0.019	0.039
Streptococcus salivarius group 1	FDSLNH	0.003	0.011	0.783	0.970	-0.018	0.024
Streptococcus salivarius group 1	DSLNH	0.002	0.032	0.942	0.970	-0.061	0.066
Streptococcus salivarius group 1	Delivery mode: emergency caesarean	1.289	3.201	0.690	0.970	-4.986	7.564
Streptococcus salivarius group 1	Delivery mode: planned caesarean	-0.484	1.920	0.803	0.970	-4.248	3.280
Streptococcus salivarius group 1	First week pacifier: yes	2.325	2.333	0.327	0.970	-2.247	6.897
Streptococcus salivarius group 1	Siblings: yes	-0.998	2.167	0.649	0.970	-5.246	3.250
Streptococcus salivarius group 1	Pre-pregnancy BMI: high and obese	-1.952	1.880	0.308	0.970	-5.637	1.733
Veillonella sp.	Secretor status: Secretor	-2.821	5.591	0.618	0.809	-13.779	8.138
Veillonella sp.	2’FL	-0.001	0.003	0.722	0.809	-0.006	0.004
Veillonella sp.	3FL	-0.008	0.007	0.245	0.809	-0.021	0.005
Veillonella sp.	DFLac	-0.006	0.011	0.602	0.809	-0.028	0.016
Veillonella sp.	3’SL	-0.009	0.024	0.714	0.809	-0.055	0.038
Veillonella sp.	6’SL	-0.022	0.024	0.372	0.809	-0.069	0.025
Veillonella sp.	LNT	-0.006	0.011	0.615	0.809	-0.027	0.016
Veillonella sp.	LNnT	-0.060	0.041	0.151	0.809	-0.141	0.020
Veillonella sp.	LNFPI	0.005	0.008	0.548	0.809	-0.011	0.021
Veillonella sp.	LNFPII	0.015	0.012	0.201	0.809	-0.008	0.038
Veillonella sp.	LNFPIII	0.348	0.383	0.372	0.809	-0.403	1.099
Veillonella sp.	LSTb	-0.040	0.070	0.570	0.809	-0.178	0.098
Veillonella sp.	LSTc	0.044	0.067	0.518	0.809	-0.087	0.175
Veillonella sp.	DFLNT	0.008	0.006	0.230	0.809	-0.005	0.021
Veillonella sp.	LNH	0.068	0.056	0.234	0.809	-0.042	0.179
Veillonella sp.	DSLNT	-0.043	0.044	0.337	0.809	-0.130	0.043
Veillonella sp.	FLNH	0.005	0.025	0.833	0.860	-0.044	0.055

Veillonella sp.	DFLNH	-0.003	0.018	0.860	0.860	-0.038	0.031
Veillonella sp.	FDSLNH	-0.008	0.013	0.545	0.809	-0.033	0.017
Veillonella sp.	DSLNH	0.015	0.038	0.693	0.809	-0.060	0.091
Veillonella sp.	Delivery mode: emergency caesarean	-2.318	3.813	0.548	0.809	-9.792	5.155
Veillonella sp.	Delivery mode: planned caesarean	1.955	2.287	0.400	0.809	-2.527	6.438
Veillonella sp.	First week pacifier: yes	-3.859	2.778	0.176	0.809	-9.304	1.586
Veillonella sp.	Siblings: yes	-0.599	2.581	0.818	0.860	-5.658	4.461
Veillonella sp.	Pre-pregnancy BMI: high and obese	-2.129	2.239	0.350	0.809	-6.518	2.260
Neisseria subflava	Secretor status: Secretor	-2.818	2.235	0.218	0.485	-7.199	1.563
Neisseria subflava	2’FL	0.000	0.001	0.882	0.882	-0.002	0.002
Neisseria subflava	3FL	-0.008	0.003	0.007	0.063	-0.013	-0.003
Neisseria subflava	DFLac	0.007	0.004	0.117	0.373	-0.002	0.016
Neisseria subflava	3’SL	0.020	0.009	0.043	0.239	0.002	0.039
Neisseria subflava	6’SL	-0.005	0.010	0.579	0.704	-0.024	0.013
Neisseria subflava	LNT	-0.007	0.004	0.130	0.373	-0.015	0.002
Neisseria subflava	LNnT	-0.028	0.016	0.096	0.373	-0.060	0.004
Neisseria subflava	LNFPI	0.001	0.003	0.864	0.882	-0.006	0.007
Neisseria subflava	LNFPII	0.016	0.005	0.002	**0.025**	0.007	0.025
Neisseria subflava	LNFPIII	-0.070	0.153	0.651	0.720	-0.370	0.230
Neisseria subflava	LSTb	0.013	0.028	0.650	0.720	-0.042	0.068
Neisseria subflava	LSTc	-0.026	0.027	0.343	0.601	-0.078	0.027
Neisseria subflava	DFLNT	0.002	0.003	0.513	0.704	-0.003	0.007
Neisseria subflava	LNH	0.052	0.022	0.028	0.197	0.008	0.096
Neisseria subflava	DSLNT	-0.019	0.018	0.280	0.561	-0.054	0.015
Neisseria subflava	FLNH	0.010	0.010	0.340	0.601	-0.010	0.030
Neisseria subflava	DFLNH	-0.004	0.007	0.579	0.704	-0.018	0.010
Neisseria subflava	FDSLNH	-0.008	0.005	0.149	0.378	-0.018	0.002
Neisseria subflava	DSLNH	0.007	0.015	0.668	0.720	-0.023	0.037
Neisseria subflava	Delivery mode: emergency caesarean	0.981	1.524	0.525	0.704	-2.007	3.968

Neisseria subflava	Delivery mode: planned caesarean	3.171	0.914	0.002	**0.025**	1.379	4.963
Neisseria subflava	First week pacifier: yes	-0.956	1.111	0.397	0.654	-3.132	1.221
Neisseria subflava	Siblings: yes	0.633	1.032	0.544	0.704	-1.389	2.656
Neisseria subflava	Pre-pregnancy BMI: high and obese	-1.707	0.895	0.067	0.312	-3.461	0.048
Neisseria subflava	Pre-pregnancy BMI: high and obese	-1.451	0.938	0.133	0.373	-3.288	0.387
Streptococcus oralis	Secretor status: Secretor	-1.835	4.926	0.712	0.986	-11.491	7.820
Streptococcus oralis	2’FL	-0.002	0.002	0.380	0.887	-0.007	0.003
Streptococcus oralis	3FL	-0.007	0.006	0.242	0.868	-0.019	0.005
Streptococcus oralis	DFLac	-0.005	0.010	0.621	0.966	-0.024	0.014
Streptococcus oralis	3’SL	0.000	0.021	0.986	0.986	-0.041	0.041
Streptococcus oralis	6’SL	-0.024	0.021	0.261	0.868	-0.066	0.017
Streptococcus oralis	LNT	-0.016	0.010	0.115	0.868	-0.034	0.003
Streptococcus oralis	LNnT	-0.035	0.036	0.341	0.868	-0.106	0.036
Streptococcus oralis	LNFPI	0.005	0.007	0.449	0.898	-0.008	0.019
Streptococcus oralis	LNFPII	0.009	0.010	0.413	0.889	-0.012	0.029
Streptococcus oralis	LNFPIII	0.081	0.338	0.813	0.986	-0.581	0.743
Streptococcus oralis	LSTb	0.065	0.062	0.302	0.868	-0.056	0.187
Streptococcus oralis	LSTc	-0.039	0.059	0.514	0.899	-0.155	0.077
Streptococcus oralis	DFLNT	0.007	0.006	0.251	0.868	-0.004	0.018
Streptococcus oralis	LNH	0.050	0.050	0.321	0.868	-0.047	0.147
Streptococcus oralis	DSLNT	-0.028	0.039	0.483	0.899	-0.104	0.049
Streptococcus oralis	FLNH	-0.005	0.022	0.831	0.986	-0.049	0.039
Streptococcus oralis	DFLNH	0.000	0.016	0.985	0.986	-0.031	0.030
Streptococcus oralis	FDSLNH	-0.001	0.011	0.954	0.986	-0.023	0.021
Streptococcus oralis	DSLNH	0.052	0.034	0.140	0.868	-0.015	0.118
Streptococcus oralis	Delivery mode: emergency caesarean	0.462	3.359	0.892	0.986	-6.123	7.046
Streptococcus oralis	Delivery mode: planned caesarean	2.307	2.015	0.262	0.868	-1.642	6.257
Streptococcus oralis	First week pacifier: yes	-1.314	2.448	0.596	0.966	-6.111	3.484

Streptococcus oralis	Siblings: yes	-0.645	2.274	0.779	0.986	-5.102	3.813
Streptococcus oralis	Pre-pregnancy BMI: high and obese	-2.489	1.973	0.218	0.868	-6.356	1.378
Haemophilus haemolyticus	Secretor status: Secretor	-4.301	3.775	0.264	0.529	-11.701	3.099
Haemophilus haemolyticus	2’FL	-0.003	0.002	0.127	0.324	-0.007	0.001
Haemophilus haemolyticus	3FL	-0.008	0.005	0.091	0.317	-0.017	0.001
Haemophilus haemolyticus	DFLac	0.006	0.008	0.447	0.630	-0.009	0.021
Haemophilus haemolyticus	3’SL	0.022	0.016	0.171	0.368	-0.009	0.054
Haemophilus haemolyticus	6’SL	0.015	0.016	0.352	0.580	-0.016	0.047
Haemophilus haemolyticus	LNT	-0.006	0.007	0.455	0.630	-0.020	0.009
Haemophilus haemolyticus	LNnT	0.013	0.028	0.646	0.772	-0.041	0.067
Haemophilus haemolyticus	LNFPI	-0.004	0.005	0.473	0.630	-0.015	0.007
Haemophilus haemolyticus	LNFPII	0.013	0.008	0.113	0.324	-0.003	0.028
Haemophilus haemolyticus	LNFPIII	0.531	0.259	0.050	0.232	0.024	1.038
Haemophilus haemolyticus	LSTb	0.002	0.048	0.965	0.965	-0.091	0.095
Haemophilus haemolyticus	LSTc	-0.046	0.045	0.321	0.580	-0.134	0.043
Haemophilus haemolyticus	DFLNT	-0.001	0.004	0.858	0.924	-0.009	0.008
Haemophilus haemolyticus	LNH	0.021	0.038	0.579	0.737	-0.053	0.096
Haemophilus haemolyticus	DSLNT	-0.086	0.030	0.007	0.152	-0.145	-0.028
Haemophilus haemolyticus	FLNH	-0.007	0.017	0.689	0.772	-0.040	0.027
Haemophilus haemolyticus	DFLNH	-0.019	0.012	0.116	0.324	-0.043	0.004
Haemophilus haemolyticus	FDSLNH	-0.021	0.009	0.022	0.152	-0.038	-0.004
Haemophilus haemolyticus	DSLNH	0.054	0.026	0.049	0.232	0.003	0.104
Haemophilus haemolyticus	Delivery mode: emergency caesarean	3.798	2.575	0.151	0.353	-1.248	8.845
Haemophilus haemolyticus	Delivery mode: planned caesarean	-2.760	1.544	0.085	0.317	-5.787	0.267
Haemophilus haemolyticus	First week pacifier: yes	4.661	1.876	0.019	0.152	0.984	8.338

Haemophilus haemolyticus	Siblings: yes	1.332	1.743	0.451	0.630	-2.085	4.748
Haemophilus haemolyticus	Pre-pregnancy BMI: high and obese	1.482	1.512	0.336	0.580	-1.482	4.446
Veillonella nakazawae	Secretor status: Secretor	-0.352	4.001	0.931	0.973	-8.194	7.491
Veillonella nakazawae	2’FL	0.002	0.002	0.244	0.872	-0.002	0.006
Veillonella nakazawae	3FL	-0.006	0.005	0.201	0.872	-0.016	0.003
Veillonella nakazawae	DFLac	-0.006	0.008	0.470	0.873	-0.022	0.010
Veillonella nakazawae	3’SL	-0.003	0.017	0.840	0.973	-0.037	0.030
Veillonella nakazawae	6’SL	-0.008	0.017	0.631	0.973	-0.042	0.025
Veillonella nakazawae	LNT	-0.005	0.008	0.499	0.873	-0.020	0.010
Veillonella nakazawae	LNnT	-0.005	0.029	0.853	0.973	-0.063	0.052
Veillonella nakazawae	LNFPI	0.002	0.006	0.749	0.973	-0.009	0.013
Veillonella nakazawae	LNFPII	0.016	0.008	0.073	0.872	-0.001	0.032
Veillonella nakazawae	LNFPIII	-0.491	0.274	0.084	0.872	-1.029	0.046
Veillonella nakazawae	LSTb	0.046	0.050	0.374	0.872	-0.053	0.144
Veillonella nakazawae	LSTc	0.018	0.048	0.704	0.973	-0.076	0.112
Veillonella nakazawae	DFLNT	0.001	0.005	0.841	0.973	-0.008	0.010
Veillonella nakazawae	LNH	-0.033	0.040	0.419	0.873	-0.112	0.046
Veillonella nakazawae	DSLNT	-0.004	0.032	0.898	0.973	-0.066	0.058
Veillonella nakazawae	FLNH	0.022	0.018	0.228	0.872	-0.013	0.058
Veillonella nakazawae	DFLNH	-0.001	0.013	0.955	0.973	-0.025	0.024
Veillonella nakazawae	FDSLNH	-0.004	0.009	0.669	0.973	-0.022	0.014
Veillonella nakazawae	DSLNH	-0.015	0.028	0.599	0.973	-0.069	0.039
Veillonella nakazawae	Delivery mode: emergency caesarean	2.813	2.729	0.311	0.872	-2.535	8.161
Veillonella nakazawae	Delivery mode: planned caesarean	2.486	1.637	0.140	0.872	-0.721	5.694
Veillonella nakazawae	First week pacifier: yes	-1.960	1.988	0.333	0.872	-5.857	1.937
Veillonella nakazawae	Siblings: yes	-0.063	1.847	0.973	0.973	-3.683	3.558
Veillonella nakazawae	Pre-pregnancy BMI: high and obese	-1.464	1.603	0.369	0.872	-4.605	1.677
Staphylococcus lugdunensis	Secretor status: Secretor	3.945	3.410	0.257	0.754	-2.738	10.627

Staphylococcus lugdunensis	2’FL	0.002	0.002	0.311	0.754	-0.002	0.005
Staphylococcus lugdunensis	3FL	0.009	0.004	0.044	0.619	0.001	0.017
Staphylococcus lugdunensis	DFLac	0.007	0.007	0.341	0.754	-0.007	0.020
Staphylococcus lugdunensis	3’SL	-0.042	0.014	0.007	0.201	-0.070	-0.014
Staphylococcus lugdunensis	6’SL	0.009	0.015	0.550	0.830	-0.020	0.037
Staphylococcus lugdunensis	LNT	0.005	0.007	0.445	0.780	-0.008	0.018
Staphylococcus lugdunensis	LNnT	0.024	0.025	0.350	0.754	-0.025	0.073
Staphylococcus lugdunensis	LNFPI	0.005	0.005	0.348	0.754	-0.005	0.014
Staphylococcus lugdunensis	LNFPII	-0.001	0.007	0.905	0.970	-0.015	0.013
Staphylococcus lugdunensis	LNFPIII	0.069	0.234	0.770	0.919	-0.389	0.527
Staphylococcus lugdunensis	LSTb	-0.054	0.043	0.221	0.754	-0.138	0.030
Staphylococcus lugdunensis	LSTc	0.010	0.041	0.806	0.919	-0.070	0.090
Staphylococcus lugdunensis	DFLNT	-0.004	0.004	0.290	0.754	-0.012	0.003
Staphylococcus lugdunensis	LNH	-0.001	0.034	0.968	0.970	-0.069	0.066
Staphylococcus lugdunensis	DSLNT	0.006	0.027	0.821	0.919	-0.047	0.059
Staphylococcus lugdunensis	FLNH	-0.001	0.015	0.970	0.970	-0.031	0.030
Staphylococcus lugdunensis	DFLNH	0.009	0.011	0.415	0.780	-0.012	0.030
Staphylococcus lugdunensis	FDSLNH	-0.004	0.008	0.634	0.846	-0.019	0.012
Staphylococcus lugdunensis	DSLNH	-0.038	0.023	0.119	0.754	-0.084	0.008
Staphylococcus lugdunensis	Delivery mode: emergency caesarean	-1.263	2.325	0.591	0.830	-5.820	3.295
Staphylococcus lugdunensis	Delivery mode: planned caesarean	-1.682	1.395	0.238	0.754	-4.415	1.052
Staphylococcus lugdunensis	First week pacifier: yes	1.230	1.694	0.474	0.780	-2.091	4.551
Staphylococcus lugdunensis	Siblings: yes	-1.537	1.574	0.337	0.754	-4.622	1.548
Staphylococcus lugdunensis	Pre-pregnancy BMI: high and obese	-0.738	1.366	0.593	0.830	-3.415	1.938
Haemophilus parainfluenzae	Secretor status: Secretor	3.187	3.725	0.400	0.721	-4.114	10.487

Haemophilus parainfluenzae	2’FL	-0.001	0.002	0.660	0.886	-0.004	0.003
Haemophilus parainfluenzae	3FL	-0.003	0.005	0.479	0.790	-0.012	0.006
Haemophilus parainfluenzae	DFLac	0.019	0.007	0.016	0.350	0.005	0.034
Haemophilus parainfluenzae	3’SL	-0.016	0.016	0.332	0.721	-0.046	0.015
Haemophilus parainfluenzae	6’SL	0.023	0.016	0.157	0.721	-0.008	0.054
Haemophilus parainfluenzae	LNT	0.005	0.007	0.527	0.819	-0.010	0.019
Haemophilus parainfluenzae	LNnT	0.026	0.027	0.350	0.721	-0.028	0.079
Haemophilus parainfluenzae	LNFPI	-0.007	0.005	0.206	0.721	-0.017	0.004
Haemophilus parainfluenzae	LNFPII	0.004	0.008	0.639	0.886	-0.012	0.019
Haemophilus parainfluenzae	LNFPIII	0.605	0.255	0.025	0.350	0.104	1.105
Haemophilus parainfluenzae	LSTb	0.010	0.047	0.835	0.886	-0.082	0.102
Haemophilus parainfluenzae	LSTc	-0.010	0.045	0.829	0.886	-0.097	0.078
Haemophilus parainfluenzae	DFLNT	-0.008	0.004	0.065	0.603	-0.017	0.000
Haemophilus parainfluenzae	LNH	-0.041	0.037	0.281	0.721	-0.115	0.032
Haemophilus parainfluenzae	DSLNT	-0.032	0.029	0.279	0.721	-0.090	0.025
Haemophilus parainfluenzae	FLNH	-0.007	0.017	0.681	0.886	-0.040	0.026
Haemophilus parainfluenzae	DFLNH	0.014	0.012	0.241	0.721	-0.009	0.037
Haemophilus parainfluenzae	FDSLNH	0.001	0.008	0.869	0.886	-0.015	0.018
Haemophilus parainfluenzae	DSLNH	-0.008	0.026	0.753	0.886	-0.058	0.042
Haemophilus parainfluenzae	Delivery mode: emergency caesarean	-3.988	2.540	0.128	0.721	-8.967	0.990
Haemophilus parainfluenzae	Delivery mode: planned caesarean	0.348	1.524	0.821	0.886	-2.639	3.334
Haemophilus parainfluenzae	First week pacifier: yes	-1.541	1.851	0.412	0.721	-5.169	2.087
Haemophilus parainfluenzae	Siblings: yes	-1.473	1.720	0.399	0.721	-4.844	1.897
Haemophilus parainfluenzae	Pre-pregnancy BMI: high and obese	1.435	1.492	0.344	0.721	-1.489	4.359
Streptococcus parasanguinis	Secretor status: Secretor	-1.784	3.864	0.648	0.824	-9.357	5.789

Streptococcus parasanguinis	2’FL	0.001	0.002	0.478	0.824	-0.002	0.005
Streptococcus parasanguinis	3FL	0.000	0.005	0.932	0.996	-0.009	0.010
Streptococcus parasanguinis	DFLac	-0.009	0.008	0.246	0.733	-0.024	0.006
Streptococcus parasanguinis	3’SL	-0.007	0.016	0.677	0.824	-0.039	0.025
Streptococcus parasanguinis	6’SL	-0.017	0.017	0.320	0.814	-0.049	0.016
Streptococcus parasanguinis	LNT	-0.011	0.007	0.137	0.733	-0.026	0.003
Streptococcus parasanguinis	LNnT	-0.032	0.028	0.262	0.733	-0.088	0.023
Streptococcus parasanguinis	LNFPI	-0.003	0.006	0.604	0.824	-0.014	0.008
Streptococcus parasanguinis	LNFPII	0.005	0.008	0.570	0.824	-0.011	0.021
Streptococcus parasanguinis	LNFPIII	-0.001	0.265	0.996	0.996	-0.520	0.518
Streptococcus parasanguinis	LSTb	0.062	0.049	0.215	0.733	-0.034	0.157
Streptococcus parasanguinis	LSTc	0.064	0.046	0.178	0.733	-0.027	0.155
Streptococcus parasanguinis	DFLNT	0.006	0.004	0.179	0.733	-0.003	0.015
Streptococcus parasanguinis	LNH	-0.034	0.039	0.387	0.824	-0.110	0.042
Streptococcus parasanguinis	DSLNT	-0.013	0.031	0.664	0.824	-0.073	0.046
Streptococcus parasanguinis	FLNH	0.040	0.018	0.031	0.433	0.005	0.074
Streptococcus parasanguinis	DFLNH	0.006	0.012	0.646	0.824	-0.018	0.030
Streptococcus parasanguinis	FDSLNH	-0.005	0.009	0.594	0.824	-0.022	0.013
Streptococcus parasanguinis	DSLNH	-0.015	0.027	0.578	0.824	-0.067	0.037
Streptococcus parasanguinis	Delivery mode: emergency caesarean	-3.326	2.635	0.217	0.733	-8.491	1.838
Streptococcus parasanguinis	Delivery mode: planned caesarean	0.063	1.580	0.969	0.996	-3.035	3.161
Streptococcus parasanguinis	First week pacifier: yes	3.047	1.920	0.124	0.733	-0.716	6.810
Streptococcus parasanguinis	Siblings: yes	-0.818	1.784	0.650	0.824	-4.314	2.678
Streptococcus parasanguinis	Pre-pregnancy BMI: high and obese	-1.055	1.547	0.501	0.824	-4.088	1.978
Bifidobacterium longum	Secretor status: Secretor	2.364	3.585	0.515	0.901	-4.662	9.391

Bifidobacterium longum	2’FL	0.000	0.002	0.996	0.996	-0.003	0.003
Bifidobacterium longum	3FL	-0.008	0.004	0.089	0.433	-0.016	0.001
Bifidobacterium longum	DFLac	-0.001	0.007	0.869	0.996	-0.015	0.013
Bifidobacterium longum	3’SL	0.028	0.015	0.075	0.433	-0.002	0.058
Bifidobacterium longum	6’SL	0.001	0.015	0.925	0.996	-0.029	0.032
Bifidobacterium longum	LNT	-0.001	0.007	0.904	0.996	-0.014	0.013
Bifidobacterium longum	LNnT	0.005	0.026	0.836	0.996	-0.046	0.057
Bifidobacterium longum	LNFPI	-0.008	0.005	0.121	0.433	-0.018	0.002
Bifidobacterium longum	LNFPII	0.002	0.008	0.829	0.996	-0.013	0.016
Bifidobacterium longum	LNFPIII	-0.269	0.246	0.283	0.683	-0.751	0.213
Bifidobacterium longum	LSTb	0.076	0.045	0.103	0.433	-0.012	0.165
Bifidobacterium longum	LSTc	-0.046	0.043	0.294	0.683	-0.130	0.038
Bifidobacterium longum	DFLNT	-0.008	0.004	0.066	0.433	-0.016	0.000
Bifidobacterium longum	LNH	0.054	0.036	0.147	0.456	-0.017	0.124
Bifidobacterium longum	DSLNT	0.056	0.028	0.058	0.433	0.001	0.112
Bifidobacterium longum	FLNH	-0.026	0.016	0.124	0.433	-0.058	0.006
Bifidobacterium longum	DFLNH	0.008	0.011	0.480	0.895	-0.014	0.030
Bifidobacterium longum	FDSLNH	0.001	0.008	0.935	0.996	-0.015	0.017
Bifidobacterium longum	DSLNH	0.044	0.025	0.085	0.433	-0.004	0.092
Bifidobacterium longum	Delivery mode: emergency caesarean	2.492	2.445	0.317	0.683	-2.300	7.283
Bifidobacterium longum	Delivery mode: planned caesarean	1.382	1.466	0.354	0.708	-1.492	4.256
Bifidobacterium longum	First week pacifier: yes	-0.358	1.781	0.842	0.996	-3.849	3.134
Bifidobacterium longum	Siblings: yes	1.818	1.655	0.281	0.683	-1.426	5.062
Bifidobacterium longum	Pre-pregnancy BMI: high and obese	-0.576	1.436	0.692	0.996	-3.390	2.239
Lactobacillus gasseri	Secretor status: Secretor	2.964	4.692	0.533	0.998	-6.233	12.161

Lactobacillus gasseri	2’FL	-0.001	0.002	0.795	0.998	-0.005	0.004
Lactobacillus gasseri	3FL	0.007	0.006	0.222	0.989	-0.004	0.018
Lactobacillus gasseri	DFLac	0.003	0.009	0.783	0.998	-0.016	0.021
Lactobacillus gasseri	3’SL	-0.007	0.020	0.711	0.998	-0.046	0.032
Lactobacillus gasseri	6’SL	-0.016	0.020	0.424	0.989	-0.056	0.023
Lactobacillus gasseri	LNT	0.000	0.009	0.979	0.998	-0.018	0.018
Lactobacillus gasseri	LNnT	0.045	0.034	0.202	0.989	-0.022	0.112
Lactobacillus gasseri	LNFPI	0.010	0.007	0.162	0.989	-0.004	0.023
Lactobacillus gasseri	LNFPII	-0.007	0.010	0.495	0.998	-0.026	0.012
Lactobacillus gasseri	LNFPIII	-0.436	0.322	0.186	0.989	-1.067	0.195
Lactobacillus gasseri	LSTb	0.000	0.059	0.996	0.998	-0.116	0.116
Lactobacillus gasseri	LSTc	-0.005	0.056	0.928	0.998	-0.115	0.105
Lactobacillus gasseri	DFLNT	-0.001	0.005	0.879	0.998	-0.011	0.010
Lactobacillus gasseri	LNH	-0.065	0.047	0.179	0.989	-0.157	0.027
Lactobacillus gasseri	DSLNT	0.036	0.037	0.334	0.989	-0.036	0.109
Lactobacillus gasseri	FLNH	0.004	0.021	0.854	0.998	-0.038	0.046
Lactobacillus gasseri	DFLNH	0.000	0.015	0.998	0.998	-0.029	0.029
Lactobacillus gasseri	FDSLNH	0.009	0.011	0.392	0.989	-0.012	0.030
Lactobacillus gasseri	DSLNH	0.002	0.032	0.951	0.998	-0.061	0.065
Lactobacillus gasseri	Delivery mode: emergency caesarean	0.311	3.200	0.923	0.998	-5.961	6.583
Lactobacillus gasseri	Delivery mode: planned caesarean	-1.962	1.919	0.315	0.989	-5.724	1.800
Lactobacillus gasseri	First week pacifier: yes	0.495	2.332	0.833	0.998	-4.075	5.065
Lactobacillus gasseri	Siblings: yes	-1.971	2.166	0.371	0.989	-6.217	2.275
Lactobacillus gasseri	Pre-pregnancy BMI: high and obese	0.110	1.879	0.954	0.998	-3.574	3.794
Porphyromonas sp.	Secretor status: Secretor	-0.189	3.215	0.954	0.967	-6.489	6.112
Porphyromonas sp.	2’FL	-0.002	0.002	0.351	0.771	-0.005	0.002
Porphyromonas sp.	3FL	-0.005	0.004	0.199	0.558	-0.013	0.003
Porphyromonas sp.	DFLac	0.009	0.006	0.154	0.558	-0.003	0.022
Porphyromonas sp.	3’SL	0.011	0.014	0.446	0.780	-0.016	0.037

Porphyromonas sp.	6’SL	0.007	0.014	0.640	0.872	-0.020	0.034
Porphyromonas sp.	LNT	-0.012	0.006	0.061	0.429	-0.024	0.000
Porphyromonas sp.	LNnT	-0.006	0.024	0.809	0.908	-0.052	0.040
Porphyromonas sp.	LNFPI	0.002	0.005	0.650	0.872	-0.007	0.011
Porphyromonas sp.	LNFPII	0.017	0.007	0.018	0.165	0.004	0.030
Porphyromonas sp.	LNFPIII	0.559	0.220	0.017	0.165	0.127	0.991
Porphyromonas sp.	LSTb	0.055	0.040	0.188	0.558	-0.025	0.134
Porphyromonas sp.	LSTc	0.017	0.039	0.654	0.872	-0.058	0.093
Porphyromonas sp.	DFLNT	0.000	0.004	0.967	0.967	-0.007	0.007
Porphyromonas sp.	LNH	-0.012	0.032	0.717	0.908	-0.075	0.051
Porphyromonas sp.	DSLNT	-0.082	0.025	0.003	0.091	-0.131	-0.032
Porphyromonas sp.	FLNH	0.023	0.015	0.121	0.558	-0.005	0.052
Porphyromonas sp.	DFLNH	-0.017	0.010	0.114	0.558	-0.036	0.003
Porphyromonas sp.	FDSLNH	-0.007	0.007	0.320	0.771	-0.022	0.007
Porphyromonas sp.	DSLNH	0.007	0.022	0.761	0.908	-0.037	0.050
Porphyromonas sp.	Delivery mode: emergency caesarean	-2.925	2.192	0.193	0.558	-7.222	1.372
Porphyromonas sp.	Delivery mode: planned caesarean	0.621	1.315	0.640	0.872	-1.956	3.198
Porphyromonas sp.	First week pacifier: yes	-0.977	1.597	0.546	0.872	-4.108	2.153
Porphyromonas sp.	Siblings: yes	-1.237	1.484	0.412	0.771	-4.145	1.672
Porphyromonas sp.	Pre-pregnancy BMI: high and obese	-1.069	1.287	0.413	0.771	-3.593	1.454

2'-fucosyllactose (2'FL), 3'-fucosyllactose (3FL), difucosyllactose (DFLac), 3'-sialyllactose (3'SL), 6'-sialyllactose (6'SL), difucosyl-N-hexosyl (DFLNH), difucosyl-N-acetyl (DFLNT), disialyl-N-hexosyl (DSLNH), disialyl-N-acetyl (DSLNT), fucosyl-disialyl-N-hexosyl (FDSLNH), fucosyllacto-N-hexaose (FLNH), lacto-N-fucopentaose I (LNFP I), lacto-N-fucopentaose II (LNFP II), lacto-N-fucopentaose III (LNFP III), lacto-N-hexaose (LNH), lacto-N-neotetraose (LNnT), lacto-N-tetraose (LNT), lacto-N-neotriose (LSTc), lacto-N-neotriose (LSTb).

Supplementary Table S7. Outputs from linear model for associations between HMO intakes and the infant oral microbiome at 2 months postpartum. Bold text indicated significant *P*-values.

**Response variable**	**Explanatory variable**	**Estimate**	**Standard Error**	***P*-value**	**BH corrected *P*-value**	**CI Lower**	**CI Upper**
Shannon diversity	2'FL	-0.0000005	0.0000004	0.211	0.634	-0.0000012 8	0.0000002 8
Shannon diversity	3FL	-0.0000015	0.0000010	0.141	0.529	-0.0000034 6	0.0000004 6
Shannon diversity	DFLac	0.0000035	0.0000016	0.048	0.344	0.0000003 6	0.0000066 4
Shannon diversity	3'SL	0.0000012	0.0000041	0.776	0.878	-0.0000068 4	0.0000092 4
Shannon diversity	6'SL	0.0000038	0.0000046	0.417	0.867	-0.0000052 2	0.0000128 2
Shannon diversity	LNT	-0.0000020	0.0000020	0.334	0.751	-0.0000059 2	0.0000019 2
Shannon diversity	LNnT	-0.0000022	0.0000060	0.716	0.878	-0.0000139 6	0.0000095 6
Shannon diversity	LNFPI	0.0000027	0.0000018	0.157	0.529	-0.0000008 3	0.0000062 3
Shannon diversity	LNFPII	0.0000023	0.0000022	0.320	0.751	-0.0000020 1	0.0000066 1
Shannon diversity	LNFPIII	-0.0001415	0.0000727	0.067	0.360	-0.0002839 9	0.0000009 9
Shannon diversity	LSTb	0.0000080	0.0000149	0.596	0.878	-0.0000212 0	0.0000372 0
Shannon diversity	LSTc	0.0000083	0.0000123	0.505	0.878	-0.0000158 1	0.0000324 1
Shannon diversity	DFLNT	-0.0000014	0.0000012	0.267	0.720	-0.0000037 5	0.0000009 5
Shannon diversity	LNH	0.0000053	0.0000091	0.567	0.878	-0.0000125 4	0.0000231 4
Shannon diversity	DSLNT	0.0000039	0.0000079	0.628	0.878	-0.0000115 8	0.0000193 8

Shannon diversity	FLNH	0.0000001	0.0000045	0.988	0.988	-0.00000872	0.00000892
Shannon diversity	DFLNH	-0.0000012	0.0000043	0.788	0.878	-0.00000963	0.00000723
Shannon diversity	FDSLNH	0.0000007	0.0000029	0.813	0.878	-0.00000498	0.00000638
Shannon diversity	DSLNH	-0.0000031	0.0000087	0.724	0.878	-0.00002015	0.00001395
Shannon diversity	Delivery mode: emergency caesarean	0.2268962	0.5072904	0.660	0.878	-0.76739298	1.22118538
Shannon diversity	Delivery mode: planned caesarean	-0.6846756	0.3285989	0.051	0.344	-1.32872944	-0.04062176
Shannon diversity	First week pacifier: yes	-0.0454418	0.3566770	0.900	0.935	-0.74452872	0.65364512
Shannon diversity	Siblings: yes	0.1088624	0.3245504	0.741	0.878	-0.52725638	0.74498118
Shannon diversity	Pre-pregnancy BMI: high and obese	-0.7039731	0.3163741	0.038	0.344	-1.32406634	-0.08387986
Richness	2’FL	-0.0000577	0.0001191	0.633	0.801	-0.00029114	0.00017574
Richness	3FL	-0.0003349	0.0002862	0.256	0.692	-0.00089585	0.00022605
Richness	DFLac	0.0013134	0.0004751	0.012	0.111	0.00038220	0.00224460
Richness	3’SL	0.0008956	0.0011963	0.463	0.750	-0.00144915	0.00324035
Richness	6’SL	0.0012708	0.0013477	0.358	0.750	-0.00137069	0.00391229
Richness	LNT	-0.0003490	0.0005818	0.556	0.775	-0.00148933	0.00079133
Richness	LNnT	-0.0006331	0.0017583	0.723	0.813	-0.00407937	0.00281317

Richness	LNFPI	0.0009297	0.0005249	0.093	0.357	-0.00009910	0.00195850
Richness	LNFPII	0.0006116	0.0006542	0.362	0.750	-0.00067063	0.00189383
Richness	LNFPIII	-0.0617149	0.0211514	0.009	0.111	-0.10317164	-0.02025816
Richness	LSTb	0.0011709	0.0043269	0.790	0.853	-0.00730982	0.00965162
Richness	LSTc	0.0016301	0.0035626	0.652	0.801	-0.00535260	0.00861280
Richness	DFLNT	-0.0008078	0.0003445	0.030	0.163	-0.00148302	-0.00013258
Richness	LNH	0.0022856	0.0026339	0.396	0.750	-0.00287684	0.00744804
Richness	DSLNT	0.0035753	0.0022952	0.136	0.458	-0.00092329	0.00807389
Richness	FLNH	-0.0000294	0.0012990	0.982	0.982	-0.00257544	0.00251664
Richness	DFLNH	-0.0010186	0.0012648	0.431	0.750	-0.00349761	0.00146041
Richness	FDSLNH	0.0001193	0.0008302	0.887	0.921	-0.00150789	0.00174649
Richness	DSLNH	-0.0017449	0.0025378	0.500	0.750	-0.00671899	0.00322919
Richness	Delivery mode: emergency caesarean	56.8358232	147.5228361	0.704	0.813	-232.30893556	345.98058196
Richness	Delivery mode: planned caesarean	-223.7984762	95.5583497	0.030	0.163	-411.09284161	-36.50411079
Richness	First week pacifier: yes	-59.3743709	103.7236100	0.574	0.775	-262.67264650	143.92390470
Richness	Siblings: yes	75.7479402	94.3810215	0.432	0.750	-109.23886194	260.73474234

Richness	Pre-pregnancy BMI: high and obese	-183.0882376	92.0033291	0.061	0.275	-363.41476264	-2.76171256
Streptococcus mitis	2’FL	0.0000006	0.0000017	0.733	0.992	-0.00000273	0.00000393
Streptococcus mitis	3FL	-0.0000031	0.0000042	0.473	0.992	-0.00001133	0.00000513
Streptococcus mitis	DFLac	-0.0000070	0.0000070	0.325	0.992	-0.00002072	0.00000672
Streptococcus mitis	3’SL	-0.0000024	0.0000175	0.891	0.992	-0.00003670	0.00003190
Streptococcus mitis	6’SL	-0.0000098	0.0000197	0.624	0.992	-0.00004841	0.00002881
Streptococcus mitis	LNT	0.0000040	0.0000085	0.643	0.992	-0.00001266	0.00002066
Streptococcus mitis	LNnT	-0.0000341	0.0000257	0.201	0.992	-0.00008447	0.00001627
Streptococcus mitis	LNFPI	-0.0000091	0.0000077	0.250	0.992	-0.00002419	0.00000599
Streptococcus mitis	LNFPII	0.0000001	0.0000096	0.992	0.992	-0.00001872	0.00001892
Streptococcus mitis	LNFPIII	0.0002270	0.0003095	0.472	0.992	-0.00037962	0.00083362
Streptococcus mitis	LSTb	-0.0000098	0.0000633	0.879	0.992	-0.00013387	0.00011427
Streptococcus mitis	LSTc	0.0000253	0.0000521	0.633	0.992	-0.00007682	0.00012742
Streptococcus mitis	DFLNT	0.0000037	0.0000050	0.470	0.992	-0.00000610	0.00001350
Streptococcus mitis	LNH	0.0000354	0.0000385	0.370	0.992	-0.00004006	0.00011086
Streptococcus mitis	DSLNT	-0.0000049	0.0000336	0.885	0.992	-0.00007076	0.00006096
Streptococcus mitis	FLNH	-0.0000113	0.0000190	0.558	0.992	-0.00004854	0.00002594

Streptococcus mitis	DFLNH	0.0000119	0.0000185	0.529	0.992	-0.00002436	0.00004816
Streptococcus mitis	FDSLNH	-0.0000004	0.0000121	0.975	0.992	-0.00002412	0.00002332
Streptococcus mitis	DSLNH	0.0000057	0.0000371	0.879	0.992	-0.00006702	0.00007842
Streptococcus mitis	Delivery mode: emergency caesarean	0.4618240	2.1585901	0.833	0.992	-3.7690126 0	4.6926606 0
Streptococcus mitis	Delivery mode: planned caesarean	1.4569592	1.3982331	0.310	0.992	-1.28357768	4.19749608
Streptococcus mitis	First week pacifier: yes	0.9124025	1.5177092	0.555	0.992	-2.06230753	3.88711253
Streptococcus mitis	Siblings: yes	-0.2216284	1.3810061	0.874	0.992	-2.92840036	2.48514356
Streptococcus mitis	Pre-pregnancy BMI: high and obese	-0.1287052	1.3462152	0.925	0.992	-2.76728699	2.50987659
Gemella haemolysans	2’FL	0.0000013	0.0000020	0.518	0.986	-0.00000262	0.00000522
Gemella haemolysans	3FL	-0.0000080	0.0000048	0.109	0.733	-0.00001741	0.00000141
Gemella haemolysans	DFLac	0.0000089	0.0000079	0.271	0.733	-0.00000658	0.00002438
Gemella haemolysans	3’SL	-0.0000107	0.0000199	0.598	0.986	-0.00004970	0.00002830
Gemella haemolysans	6’SL	-0.0000002	0.0000224	0.994	0.994	-0.00004410	0.00004370
Gemella haemolysans	LNT	-0.0000035	0.0000097	0.720	0.986	-0.00002251	0.00001551
Gemella haemolysans	LNnT	-0.0000102	0.0000292	0.731	0.986	-0.00006743	0.00004703
Gemella haemolysans	LNFPI	-0.0000022	0.0000087	0.805	0.988	-0.00001925	0.00001485

Gemella haemolysans	LNFPII	0.0000219	0.0000109	0.058	0.733	0.00000054	0.00004326
Gemella haemolysans	LNFPIII	0.0000585	0.0003512	0.870	0.994	-0.00062985	0.00074685
Gemella haemolysans	LSTb	-0.0000062	0.0000718	0.932	0.994	-0.00014693	0.00013453
Gemella haemolysans	LSTc	-0.0000232	0.0000592	0.699	0.986	-0.00013923	0.00009283
Gemella haemolysans	DFLNT	-0.0000059	0.0000057	0.316	0.776	-0.00001707	0.00000527
Gemella haemolysans	LNH	0.0000333	0.0000437	0.456	0.946	-0.00005235	0.00011895
Gemella haemolysans	DSLNT	-0.0000518	0.0000381	0.190	0.733	-0.00012648	0.00002288
Gemella haemolysans	FLNH	0.0000091	0.0000216	0.679	0.986	-0.00003324	0.00005144
Gemella haemolysans	DFLNH	-0.0000256	0.0000210	0.239	0.733	-0.00006676	0.00001556
Gemella haemolysans	FDSLNH	-0.0000182	0.0000138	0.202	0.733	-0.00004525	0.00000885
Gemella haemolysans	DSLNH	0.0000202	0.0000421	0.637	0.986	-0.00006232	0.00010272
Gemella haemolysans	Delivery mode: emergency caesarean	2.2139593	2.4494237	0.377	0.849	-2.58691115	7.01482975
Gemella haemolysans	Delivery mode: planned caesarean	-0.0441944	1.5866214	0.978	0.994	-3.15397234	3.06558354
Gemella haemolysans	First week pacifier: yes	0.4665009	1.7221949	0.789	0.988	-2.90900110	3.84200290
Gemella haemolysans	Siblings: yes	2.5431137	1.5670734	0.121	0.733	-0.52835016	5.61457756
Gemella haemolysans	Pre-pregnancy BMI: high and obese	0.0248537	1.5275949	0.987	0.994	-2.96923230	3.01893970

Rothia mucilaginosa	2’FL	0.0000013	0.0000021	0.557	0.716	-0.0000282	0.0000542
Rothia mucilaginosa	3FL	-0.0000048	0.0000050	0.349	0.595	-0.0000146 0	0.0000050 0
Rothia mucilaginosa	DFLac	-0.0000115	0.0000084	0.184	0.451	-0.0000279 6	0.0000049 6
Rothia mucilaginosa	3’SL	-0.0000271	0.0000211	0.214	0.482	-0.0000684 6	0.0000142 6
Rothia mucilaginosa	6’SL	0.0000081	0.0000237	0.737	0.865	-0.0000383 5	0.0000545 5
Rothia mucilaginosa	LNT	-0.0000147	0.0000102	0.166	0.451	-0.0000346 9	0.0000052 9
Rothia mucilaginosa	LNnT	-0.0000059	0.0000310	0.852	0.932	-0.0000666 6	0.0000548 6
Rothia mucilaginosa	LNFPI	-0.0000214	0.0000092	0.032	0.285	-0.0000394 3	-0.0000033 7
Rothia mucilaginosa	LNFPII	-0.0000096	0.0000115	0.413	0.656	-0.0000321 4	0.0000129 4
Rothia mucilaginosa	LNFPIII	0.0006052	0.0003724	0.121	0.451	-0.0001247 0	0.0013351 0
Rothia mucilaginosa	LSTb	0.0001980	0.0000762	0.018	0.238	0.0000486 5	0.0003473 5
Rothia mucilaginosa	LSTc	0.0000214	0.0000627	0.736	0.865	-0.0001014 9	0.0001442 9
Rothia mucilaginosa	DFLNT	0.0000122	0.0000061	0.059	0.364	0.0000002 4	0.0000241 6
Rothia mucilaginosa	LNH	-0.0000657	0.0000464	0.173	0.451	-0.0001566 4	0.0000252 4
Rothia mucilaginosa	DSLNT	-0.0000385	0.0000404	0.353	0.595	-0.0001176 8	0.0000406 8
Rothia mucilaginosa	FLNH	-0.0000016	0.0000229	0.945	0.945	-0.0000464 8	0.0000432 8
Rothia mucilaginosa	DFLNH	0.0000274	0.0000223	0.233	0.484	-0.0000163 1	0.0000711 1
Rothia mucilaginosa	FDSLNH	0.0000216	0.0000146	0.156	0.451	-0.0000070 2	0.0000502 2

Rothia mucilaginosa	DSLNH	-0.0000078	0.0000447	0.863	0.932	-0.0000954 1	0.0000798 1
Rothia mucilaginosa	Delivery mode: emergency caesarean	-1.6300222	2.5972424	0.538	0.716	-6.7206173 0	3.4605729 0
Rothia mucilaginosa	Delivery mode: planned caesarean	3.2628537	1.6823714	0.067	0.364	-0.0345942 4	6.5603016 4
Rothia mucilaginosa	First week pacifier: yes	1.1920109	1.8261265	0.522	0.716	-2.3871970 4	4.7712188 4
Rothia mucilaginosa	Siblings: yes	0.1863282	1.6616437	0.912	0.945	-3.0704934 5	3.4431498 5
Rothia mucilaginosa	Pre-pregnancy BMI: high and obese	2.2538850	1.6197828	0.180	0.451	-0.9208892 9	5.4286592 9
Streptococcus salivarius group 1	2’FL	0.0000007	0.0000029	0.824	0.963	-0.0000049 8	0.0000063 8
Streptococcus salivarius group 1	3FL	0.0000120	0.0000070	0.104	0.563	-0.0000017 2	0.0000257 2
Streptococcus salivarius group 1	DFLac	-0.0000049	0.0000117	0.676	0.963	-0.0000278 3	0.0000180 3
Streptococcus salivarius group 1	3’SL	-0.0000461	0.0000293	0.132	0.596	-0.0001035 3	0.0000113 3
Streptococcus salivarius group 1	6’SL	-0.0000298	0.0000331	0.379	0.929	-0.0000946 8	0.0000350 8
Streptococcus salivarius group 1	LNT	-0.0000046	0.0000143	0.749	0.963	-0.0000326 3	0.0000234 3
Streptococcus salivarius group 1	LNnT	0.0000757	0.0000431	0.095	0.563	-0.0000087 8	0.0001601 8

Streptococcus salivarius group 1	LNFPI	0.0000119	0.0000129	0.366	0.929	-0.0000133 8	0.0000371 8
Streptococcus salivarius group 1	LNFPII	0.0000012	0.0000160	0.943	0.963	-0.0000301 6	0.0000325 6
Streptococcus salivarius group 1	LNFPIII	-0.0005037	0.0005189	0.344	0.929	-0.0015207 4	0.0005133 4
Streptococcus salivarius group 1	LSTb	0.0000287	0.0001061	0.789	0.963	-0.0001792 6	0.0002366 6
Streptococcus salivarius group 1	LSTc	0.0000042	0.0000874	0.963	0.963	-0.0001671 0	0.0001755 0
Streptococcus salivarius group 1	DFLNT	0.0000015	0.0000085	0.865	0.963	-0.0000151 6	0.0000181 6
Streptococcus salivarius group 1	LNH	-0.0001810	0.0000646	0.011	0.307	-0.0003076 2	-0.0000543 8
Streptococcus salivarius group 1	DSLNT	-0.0000333	0.0000563	0.561	0.963	-0.0001436 5	0.0000770 5
Streptococcus salivarius group 1	FLNH	0.0000608	0.0000319	0.072	0.563	-0.0000017 2	0.0001233 2
Streptococcus salivarius group 1	DFLNH	-0.0000216	0.0000310	0.495	0.963	-0.0000823 6	0.0000391 6
Streptococcus salivarius group 1	FDSLNH	-0.0000212	0.0000204	0.312	0.929	-0.0000611 8	0.0000187 8
Streptococcus salivarius group 1	DSLNH	0.0000446	0.0000623	0.482	0.963	-0.0000775 1	0.0001667 1
Streptococcus salivarius group 1	Delivery mode: emergency caesarean	1.5546627	3.6188724	0.672	0.963	-5.5383272 0	8.6476526 0

Streptococcus salivarius group 1	Delivery mode: planned caesarean	0.2098152	2.3441352	0.930	0.963	-4.3846897 9	4.80432019
Streptococcus salivarius group 1	First week pacifier: yes	-0.3267557	2.5444366	0.899	0.963	-5.3138514 4	4.66034004
Streptococcus salivarius group 1	Siblings: yes	-1.3461600	2.3152542	0.568	0.963	-5.8840582 3	3.19173823
Streptococcus salivarius group 1	Pre-pregnancy BMI: high and obese	-2.7495370	2.2569272	0.238	0.918	-7.1731143 1	1.67404031
Veillonella sp.	2’FL	-0.0000014	0.0000036	0.702	0.907	-0.00000846	0.00000566
Veillonella sp.	3FL	-0.0000128	0.0000088	0.160	0.907	-0.00003005	0.00000445
Veillonella sp.	DFLac	-0.0000049	0.0000145	0.739	0.907	-0.00003332	0.00002352
Veillonella sp.	3’SL	-0.0000035	0.0000366	0.924	0.998	-0.00007524	0.00006824
Veillonella sp.	6’SL	-0.0000313	0.0000412	0.458	0.907	-0.00011205	0.00004945
Veillonella sp.	LNT	-0.0000162	0.0000178	0.373	0.907	-0.00005109	0.00001869
Veillonella sp.	LNnT	-0.0001099	0.0000538	0.055	0.745	-0.00021535	-0.00000445
Veillonella sp.	LNFPI	0.0000040	0.0000161	0.804	0.944	-0.00002756	0.00003556
Veillonella sp.	LNFPII	0.0000200	0.0000200	0.331	0.907	-0.00001920	0.00005920
Veillonella sp.	LNFPIII	0.0006128	0.0006469	0.355	0.907	-0.00065512	0.00188072
Veillonella sp.	LSTb	0.0000460	0.0001323	0.732	0.907	-0.00021331	0.00030531
Veillonella sp.	LSTc	0.0000524	0.0001090	0.636	0.907	-0.00016124	0.00026604

Veillonella sp.	DFLNT	0.0000052	0.0000105	0.628	0.907	-0.0000153 8	0.0000257 8
Veillonella sp.	LNH	0.0001201	0.0000806	0.152	0.907	-0.0000378 8	0.0002780 8
Veillonella sp.	DSLNT	-0.0000519	0.0000702	0.468	0.907	-0.0001894 9	0.0000856 9
Veillonella sp.	FLNH	-0.0000009	0.0000397	0.982	0.999	-0.0000787 1	0.0000769 1
Veillonella sp.	DFLNH	0.0000000	0.0000387	0.999	0.999	-0.0000758 5	0.0000758 5
Veillonella sp.	FDSLNH	-0.0000044	0.0000254	0.865	0.973	-0.0000541 8	0.0000453 8
Veillonella sp.	DSLNH	0.0000369	0.0000776	0.640	0.907	-0.0001152 0	0.0001890 0
Veillonella sp.	Delivery mode: emergency caesarean	-2.1485700	4.5120259	0.639	0.907	-10.992140 76	6.6950007 6
Veillonella sp.	Delivery mode: planned caesarean	2.4290515	2.9226780	0.416	0.907	-3.2993973 8	8.1575003 8
Veillonella sp.	First week pacifier: yes	-4.0210556	3.1724147	0.220	0.907	-10.238988 41	2.1968772 1
Veillonella sp.	Siblings: yes	0.9770955	2.8866691	0.739	0.907	-4.6807759 4	6.6349669 4
Veillonella sp.	Pre-pregnancy BMI: high and obese	-1.7256989	2.8139468	0.547	0.907	-7.2410346 3	3.7896368 3
Neisseria subflava	2’FL	-0.0000004	0.0000014	0.772	0.802	-0.0000031 4	0.0000023 4
Neisseria subflava	3FL	-0.0000089	0.0000033	0.015	0.131	-0.0000153 7	-0.0000024 3
Neisseria subflava	DFLac	0.0000033	0.0000055	0.558	0.675	-0.0000074 8	0.0000140 8
Neisseria subflava	3’SL	0.0000258	0.0000138	0.077	0.260	-0.0000012 5	0.0000528 5

Neisseria subflava	6’SL	-0.0000250	0.0000156	0.124	0.305	-0.0000558	0.0000055 8
Neisseria subflava	LNT	-0.0000153	0.0000067	0.035	0.187	-0.0000284 3	-0.0000021 7
Neisseria subflava	LNnT	-0.0000462	0.0000203	0.035	0.187	-0.0000859 9	-0.0000064 1
Neisseria subflava	LNFPI	0.0000048	0.0000061	0.434	0.616	-0.0000071 6	0.0000167 6
Neisseria subflava	LNFPII	0.000030	0.0000076	0.002	**0.025**	0.0000051 0	0.0000349 0
Neisseria subflava	LNFPIII	-0.0000588	0.0002441	0.812	0.812	-0.0005372 4	0.0004196 4
Neisseria subflava	LSTb	0.0000439	0.0000499	0.391	0.586	-0.0000539 0	0.0001417 0
Neisseria subflava	LSTc	-0.0000574	0.0000411	0.179	0.322	-0.0001379 6	0.0000231 6
Neisseria subflava	DFLNT	0.0000027	0.0000040	0.506	0.650	-0.0000051 4	0.0000105 4
Neisseria subflava	LNH	0.0000546	0.0000304	0.088	0.260	-0.0000049 8	0.0001141 8
Neisseria subflava	DSLNT	-0.0000381	0.0000265	0.166	0.321	-0.0000900 4	0.0000138 4
Neisseria subflava	FLNH	0.0000274	0.0000150	0.083	0.260	-0.0000020 0	0.0000568 0
Neisseria subflava	DFLNH	-0.0000173	0.0000146	0.250	0.397	-0.0000459 2	0.0000113 2
Neisseria subflava	FDSLNH	-0.0000168	0.0000096	0.096	0.260	-0.0000356 2	0.0000020 2
Neisseria subflava	DSLNH	0.0000361	0.0000293	0.233	0.393	-0.0000213 3	0.0000935 3
Neisseria subflava	Delivery mode: emergency caesarean	0.7615988	1.7027106	0.660	0.742	-2.5757139 8	4.0989115 8
Neisseria subflava	Delivery mode: planned caesarean	4.0637724	1.1029358	0.002	**0.025**	1.9020182 3	6.2255265 7

Neisseria subflava	First week pacifier: yes	-1.7462570	1.1971793	0.161	0.321	-4.09272843	0.60021443
Neisseria subflava	Siblings: yes	0.8085570	1.0893470	0.467	0.630	-1.32656312	2.94367712
Neisseria subflava	Pre-pregnancy BMI: high and obese	-2.1463525	1.0619037	0.058	0.259	-4.22768375	-0.06502125
Streptococcus oralis	2’FL	-0.0000015	0.0000031	0.629	0.898	-0.00000758	0.00000458
Streptococcus oralis	3FL	-0.0000121	0.0000074	0.120	0.840	-0.00002660	0.00000240
Streptococcus oralis	DFLac	-0.0000090	0.0000123	0.471	0.898	-0.00003311	0.00001511
Streptococcus oralis	3’SL	-0.0000142	0.0000310	0.652	0.898	-0.00007496	0.00004656
Streptococcus oralis	6’SL	0.0000069	0.0000349	0.846	0.898	-0.00006150	0.00007530
Streptococcus oralis	LNT	-0.0000159	0.0000151	0.306	0.840	-0.00004550	0.00001370
Streptococcus oralis	LNnT	-0.0000529	0.0000455	0.260	0.840	-0.00014208	0.00003628
Streptococcus oralis	LNFPI	-0.0000069	0.0000136	0.620	0.898	-0.00003356	0.00001976
Streptococcus oralis	LNFPII	0.0000022	0.0000169	0.898	0.898	-0.00003092	0.00003532
Streptococcus oralis	LNFPIII	0.0005992	0.0005475	0.287	0.840	-0.00047390	0.00167230
Streptococcus oralis	LSTb	0.0001042	0.0001120	0.364	0.840	-0.00011532	0.00032372
Streptococcus oralis	LSTc	-0.0000306	0.0000922	0.744	0.898	-0.00021131	0.00015011
Streptococcus oralis	DFLNT	0.0000139	0.0000089	0.134	0.840	-0.00000354	0.00003134
Streptococcus oralis	LNH	0.0000622	0.0000682	0.373	0.840	-0.00007147	0.00019587

Streptococcus oralis	DSLNT	-0.0000294	0.0000594	0.626	0.898	-0.00014582	0.00008702
Streptococcus oralis	FLNH	-0.0000343	0.0000336	0.320	0.840	-0.00010016	0.00003156
Streptococcus oralis	DFLNH	0.0000346	0.0000327	0.304	0.840	-0.00002949	0.00009869
Streptococcus oralis	FDSLNH	0.0000263	0.0000215	0.236	0.840	-0.00001584	0.00006844
Streptococcus oralis	DSLNH	0.0000093	0.0000657	0.889	0.898	-0.00011947	0.00013807
Streptococcus oralis	Delivery mode: emergency caesarean	-2.3290833	3.8182682	0.549	0.898	-9.81288897	5.15472237
Streptococcus oralis	Delivery mode: planned caesarean	3.0746088	2.4732944	0.229	0.840	-1.77304822	7.92226582
Streptococcus oralis	First week pacifier: yes	-0.6079735	2.6846323	0.823	0.898	-5.86985281	4.65390581
Streptococcus oralis	Siblings: yes	-1.0420443	2.4428222	0.674	0.898	-5.82997581	3.74588721
Streptococcus oralis	Pre-pregnancy BMI: high and obese	-1.1145456	2.3812814	0.645	0.898	-5.78185714	3.55276594
Haemophilus haemolyticus	2’FL	-0.0000007	0.0000026	0.777	0.792	-0.00000580	0.00000440
Haemophilus haemolyticus	3FL	-0.0000069	0.0000063	0.283	0.551	-0.00001925	0.00000545
Haemophilus haemolyticus	DFLac	0.0000136	0.0000104	0.206	0.551	-0.00000678	0.00003398
Haemophilus haemolyticus	3’SL	0.0000229	0.0000262	0.393	0.589	-0.00002845	0.00007425

Haemophilus haemolyticus	6’SL	0.0000230	0.0000295	0.444	0.631	-0.00003482	0.00008082
Haemophilus haemolyticus	LNT	-0.0000145	0.0000127	0.267	0.551	-0.00003939	0.00001039
Haemophilus haemolyticus	LNnT	0.0000125	0.0000384	0.748	0.792	-0.00006276	0.00008776
Haemophilus haemolyticus	LNFPI	-0.0000043	0.0000115	0.713	0.792	-0.00002684	0.00001824
Haemophilus haemolyticus	LNFPII	0.0000192	0.0000143	0.195	0.551	-0.00000883	0.00004723
Haemophilus haemolyticus	LNFPIII	0.0006742	0.0004624	0.161	0.551	-0.00023210	0.00158050
Haemophilus haemolyticus	LSTb	0.0000900	0.0000946	0.353	0.589	-0.00009542	0.00027542
Haemophilus haemolyticus	LSTc	-0.0000353	0.0000779	0.655	0.792	-0.00018798	0.00011738
Haemophilus haemolyticus	DFLNT	-0.0000095	0.0000075	0.223	0.551	-0.00002420	0.00000520
Haemophilus haemolyticus	LNH	0.0000154	0.0000576	0.792	0.792	-0.00009750	0.00012830
Haemophilus haemolyticus	DSLNT	-0.0001009	0.0000502	0.059	0.551	-0.00019929	-0.00000251
Haemophilus haemolyticus	FLNH	0.0000103	0.0000284	0.720	0.792	-0.00004536	0.00006596
Haemophilus haemolyticus	DFLNH	-0.0000291	0.0000276	0.305	0.551	-0.00008320	0.00002500

Haemophilus haemolyticus	FDSLNH	-0.0000210	0.0000181	0.261	0.551	-0.0000564 8	0.0000144 8
Haemophilus haemolyticus	DSLNH	0.0000491	0.0000555	0.387	0.589	-0.0000596 8	0.0001578 8
Haemophilus haemolyticus	Delivery mode: emergency caesarean	3.3913087	3.2250333	0.306	0.551	-2.9297565 7	9.7123739 7
Haemophilus haemolyticus	Delivery mode: planned caesarean	-3.1313643	2.0890248	0.150	0.551	-7.2258529 1	0.9631243 1
Haemophilus haemolyticus	First week pacifier: yes	3.7210598	2.2675275	0.117	0.551	-0.7232941 0	8.1654137 0
Haemophilus haemolyticus	Siblings: yes	2.6991674	2.0632869	0.206	0.551	-1.3448749 2	6.7432097 2
Haemophilus haemolyticus	Pre-pregnancy BMI: high and obese	0.8553402	2.0113076	0.675	0.792	-3.0868227 0	4.7975031 0
Haemophilus haemolyticus	Pre-pregnancy BMI: high and obese	1.0576293	1.9618060	0.596	0.792	-2.7875104 6	4.9027690 6
Veillonella nakazawae	2’FL	0.0000032	0.0000023	0.194	0.309	-0.0000013 1	0.0000077 1
Veillonella nakazawae	3FL	-0.0000090	0.0000056	0.126	0.309	-0.0000199 8	0.0000019 8
Veillonella nakazawae	DFLac	-0.0000056	0.0000093	0.553	0.622	-0.0000238 3	0.0000126 3
Veillonella nakazawae	3’SL	0.0000162	0.0000235	0.500	0.622	-0.0000298 6	0.0000622 6
Veillonella nakazawae	6’SL	-0.0000363	0.0000265	0.187	0.309	-0.0000882 4	0.0000156 4
Veillonella nakazawae	LNT	-0.0000197	0.0000114	0.101	0.309	-0.0000420 4	0.0000026 4

Veillonella nakazawae	LNnT	-0.0000078	0.0000346	0.825	0.857	-0.00007562	0.00006002
Veillonella nakazawae	LNFPI	0.0000150	0.0000103	0.163	0.309	-0.00000519	0.00003519
Veillonella nakazawae	LNFPII	0.0000379	0.0000129	0.008	0.225	0.00001262	0.00006318
Veillonella nakazawae	LNFPIII	-0.0009108	0.0004158	0.041	0.244	-0.00172577	-0.00009583
Veillonella nakazawae	LSTb	0.0001158	0.0000851	0.189	0.309	-0.00005100	0.00028260
Veillonella nakazawae	LSTc	-0.0000650	0.0000700	0.365	0.517	-0.00020220	0.00007220
Veillonella nakazawae	DFLNT	-0.0000010	0.0000068	0.890	0.890	-0.00001433	0.00001233
Veillonella nakazawae	LNH	-0.0000326	0.0000518	0.536	0.622	-0.00013413	0.00006893
Veillonella nakazawae	DSLNT	-0.0000470	0.0000451	0.311	0.466	-0.00013540	0.00004140
Veillonella nakazawae	FLNH	0.0000550	0.0000255	0.044	0.244	0.00000502	0.00010498
Veillonella nakazawae	DFLNH	-0.0000400	0.0000249	0.124	0.309	-0.00008880	0.00000880
Veillonella nakazawae	FDSLNH	-0.0000263	0.0000163	0.124	0.309	-0.00005825	0.00000565
Veillonella nakazawae	DSLNH	0.0000446	0.0000499	0.383	0.517	-0.00005320	0.00014240
Veillonella nakazawae	Delivery mode: emergency caesarean	4.2372626	2.8998111	0.160	0.309	-1.44636716	9.92089236
Veillonella nakazawae	Delivery mode: planned caesarean	4.0270546	1.8783612	0.045	0.244	0.34546665	7.70864255
Veillonella nakazawae	First week pacifier: yes	-3.7554864	2.0388632	0.081	0.309	-7.75165827	0.24068547
Veillonella nakazawae	Siblings: yes	-0.4788027	1.8552188	0.799	0.857	-4.11503155	3.15742615

Veillonella nakazawae	Pre-pregnancy BMI: high and obese	-2.8744364	1.8084812	0.128	0.309	-6.41905955	0.67018675
Staphylococcus lugdunensis	2’FL	-0.0000003	0.0000017	0.875	0.936	-0.00000363	0.00000303
Staphylococcus lugdunensis	3FL	0.0000133	0.0000041	0.004	0.111	0.00000526	0.00002134
Staphylococcus lugdunensis	DFLac	0.0000083	0.0000068	0.236	0.578	-0.00000503	0.00002163
Staphylococcus lugdunensis	3’SL	-0.0000400	0.0000170	0.030	0.231	-0.00007332	-0.00000668
Staphylococcus lugdunensis	6’SL	-0.0000147	0.0000192	0.452	0.734	-0.00005233	0.00002293
Staphylococcus lugdunensis	LNT	0.0000061	0.0000083	0.468	0.734	-0.00001017	0.00002237
Staphylococcus lugdunensis	LNnT	0.0000307	0.0000250	0.235	0.578	-0.00001830	0.00007970
Staphylococcus lugdunensis	LNFPI	0.0000137	0.0000075	0.082	0.318	-0.00000100	0.00002840
Staphylococcus lugdunensis	LNFPII	0.0000012	0.0000093	0.901	0.936	-0.00001703	0.00001943
Staphylococcus lugdunensis	LNFPIII	-0.0001613	0.0003011	0.598	0.734	-0.00075146	0.00042886
Staphylococcus lugdunensis	LSTb	-0.0001407	0.0000616	0.034	0.231	-0.00026144	-0.00001996
Staphylococcus lugdunensis	LSTc	0.0000209	0.0000507	0.684	0.783	-0.00007847	0.00012027
Staphylococcus lugdunensis	DFLNT	-0.0000068	0.0000049	0.181	0.544	-0.00001640	0.00000280
Staphylococcus lugdunensis	LNH	0.0000409	0.0000375	0.288	0.598	-0.00003260	0.00011440
Staphylococcus lugdunensis	DSLNT	0.0000198	0.0000327	0.552	0.734	-0.00004429	0.00008389
Staphylococcus lugdunensis	FLNH	0.0000103	0.0000185	0.583	0.734	-0.00002596	0.00004656

Staphylococcus lugdunensis	DFLNH	-0.0000102	0.0000180	0.576	0.734	-0.0000454 8	0.0000250 8
Staphylococcus lugdunensis	FDSLNH	-0.0000256	0.0000118	0.043	0.231	-0.0000487 3	-0.0000024 7
Staphylococcus lugdunensis	DSLNH	-0.0000240	0.0000361	0.515	0.734	-0.0000947 6	0.0000467 6
Staphylococcus lugdunensis	Delivery mode: emergency caesarean	-0.0476525	2.0998817	0.982	0.982	-4.1634206 3	4.0681156 3
Staphylococcus lugdunensis	Delivery mode: planned caesarean	-3.0665587	1.3602045	0.036	0.231	-5.7325595 2	-0.4005578 8
Staphylococcus lugdunensis	First week pacifier: yes	1.5393752	1.4764312	0.310	0.598	-1.3544299 5	4.4331803 5
Staphylococcus lugdunensis	Siblings: yes	-1.5147583	1.3434461	0.274	0.598	-4.1479126 6	1.1183960 6
Staphylococcus lugdunensis	Pre-pregnancy BMI: high and obese	-1.2635407	1.3096014	0.347	0.624	-3.8303594 4	1.3032780 4
Haemophilus parainfluenzae	2’FL	0.0000002	0.0000027	0.941	0.978	-0.0000050 9	0.0000054 9
Haemophilus parainfluenzae	3FL	-0.0000043	0.0000064	0.511	0.883	-0.0000168 4	0.0000082 4
Haemophilus parainfluenzae	DFLac	0.0000170	0.0000107	0.127	0.883	-0.0000039 7	0.0000379 7
Haemophilus parainfluenzae	3’SL	-0.0000181	0.0000269	0.508	0.883	-0.0000708 2	0.0000346 2
Haemophilus parainfluenzae	6’SL	0.0000245	0.0000303	0.429	0.883	-0.0000348 9	0.0000838 9
Haemophilus parainfluenzae	LNT	0.0000128	0.0000131	0.338	0.883	-0.0000128 8	0.0000384 8

Haemophilus parainfluenzae	LNnT	0.0000302	0.0000395	0.454	0.883	-0.0000472 2	0.0001076 2
Haemophilus parainfluenzae	LNFPI	-0.0000122	0.0000118	0.315	0.883	-0.0000353 3	0.0000109 3
Haemophilus parainfluenzae	LNFPII	-0.0000004	0.0000147	0.978	0.978	-0.0000292 1	0.0000284 1
Haemophilus parainfluenzae	LNFPIII	0.0005588	0.0004754	0.254	0.883	-0.0003729 8	0.0014905 8
Haemophilus parainfluenzae	LSTb	-0.0000227	0.0000972	0.818	0.947	-0.0002132 1	0.0001678 1
Haemophilus parainfluenzae	LSTc	0.0000157	0.0000801	0.846	0.947	-0.0001413 0	0.0001727 0
Haemophilus parainfluenzae	DFLNT	-0.0000067	0.0000077	0.394	0.883	-0.0000217 9	0.0000083 9
Haemophilus parainfluenzae	LNH	-0.0000531	0.0000592	0.381	0.883	-0.0001691 3	0.0000629 3
Haemophilus parainfluenzae	DSLNT	-0.0000305	0.0000516	0.561	0.883	-0.0001316 4	0.0000706 4
Haemophilus parainfluenzae	FLNH	-0.0000133	0.0000292	0.654	0.883	-0.0000705 3	0.0000439 3
Haemophilus parainfluenzae	DFLNH	0.0000225	0.0000284	0.439	0.883	-0.0000331 6	0.0000781 6
Haemophilus parainfluenzae	FDSLNH	0.0000062	0.0000187	0.742	0.947	-0.0000304 5	0.0000428 5
Haemophilus parainfluenzae	DSLNH	-0.0000142	0.0000570	0.806	0.947	-0.0001259 2	0.0000975 2

Haemophilus parainfluenzae	Delivery mode: emergency caesarean	-3.5942817	3.3154903	0.292	0.883	-10.09264269	2.9040792 9
Haemophilus parainfluenzae	Delivery mode: planned caesarean	1.0665284	2.1476185	0.625	0.883	-3.1428038 6	5.2758606 6
Haemophilus parainfluenzae	First week pacifier: yes	-1.1617509	2.3311280	0.624	0.883	-5.7307617 8	3.4072599 8
Haemophilus parainfluenzae	Siblings: yes	-1.0622326	2.1211588	0.622	0.883	-5.2197038 5	3.0952386 5
Haemophilus parainfluenzae	Pre-pregnancy BMI: high and obese	1.4956279	2.0677215	0.478	0.883	-2.5571062 4	5.5483620 4
Streptococcus parasanguinis	2’FL	-0.0000010	0.0000018	0.597	0.767	-0.0000045 3	0.0000025 3
Streptococcus parasanguinis	3FL	-0.0000014	0.0000044	0.758	0.890	-0.0000100 2	0.0000072 2
Streptococcus parasanguinis	DFLac	-0.0000171	0.0000073	0.030	0.090	-0.0000314 1	-0.0000027 9
Streptococcus parasanguinis	3’SL	-0.0000442	0.0000184	0.027	0.090	-0.0000802 6	-0.0000081 4
Streptococcus parasanguinis	6’SL	0.0000155	0.0000207	0.464	0.627	-0.0000250 7	0.0000560 7
Streptococcus parasanguinis	LNT	0.0000017	0.0000089	0.855	0.962	-0.0000157 4	0.0000191 4
Streptococcus parasanguinis	LNnT	-0.0000447	0.0000270	0.114	0.221	-0.0000976 2	0.0000082 2
Streptococcus parasanguinis	LNFPI	-0.0000184	0.0000081	0.034	0.093	-0.0000342 8	-0.0000025 2

Streptococcus parasanguinis	LNFPII	-0.0000147	0.0000100	0.160	0.254	-0.00003430	0.00000490
Streptococcus parasanguinis	LNFPIII	0.0004752	0.0003249	0.160	0.254	-0.00016160	0.00111200
Streptococcus parasanguinis	LSTb	0.0000093	0.0000665	0.890	0.962	-0.00012104	0.00013964
Streptococcus parasanguinis	LSTc	0.0001464	0.0000547	0.015	0.081	0.00003919	0.00025361
Streptococcus parasanguinis	DFLNT	0.0000188	0.0000053	0.002	**0.028**	0.00000841	0.00002919
Streptococcus parasanguinis	LNH	-0.0000679	0.0000405	0.110	0.221	-0.00014728	0.00001148
Streptococcus parasanguinis	DSLNT	-0.0000148	0.0000353	0.678	0.833	-0.00008399	0.00005439
Streptococcus parasanguinis	FLNH	-0.0000003	0.0000200	0.988	0.988	-0.00003950	0.00003890
Streptococcus parasanguinis	DFLNH	0.0000500	0.0000194	0.006	**0.040**	0.00002198	0.00009802
Streptococcus parasanguinis	FDSLNH	0.0000203	0.0000128	0.129	0.232	-0.00000479	0.00004539
Streptococcus parasanguinis	DSLNH	-0.0000922	0.0000390	0.029	0.090	-0.00016864	-0.00001576
Streptococcus parasanguinis	Delivery mode: emergency caesarean	-7.2978526	2.2661670	0.005	**0.041**	-11.73953992	-2.85616528
Streptococcus parasanguinis	Delivery mode: planned caesarean	-1.6547183	1.4679164	0.274	0.389	-4.53183444	1.22239784

Streptococcus parasanguinis	First week pacifier: yes	4.1171748	1.5933467	0.018	0.082	0.99421527	7.24013433
Streptococcus parasanguinis	Siblings: yes	-2.7112913	1.4498309	0.077	0.189	-5.55295986	0.13037726
Streptococcus parasanguinis	Pre-pregnancy BMI: high and obese	-0.1196287	1.4133060	0.933	0.969	-2.88970846	2.65045106
Bifidobacterium longum	2’FL	-0.0000004	0.0000016	0.824	0.906	-0.00000354	0.00000274
Bifidobacterium longum	3FL	-0.0000041	0.0000037	0.286	0.455	-0.00001135	0.00000315
Bifidobacterium longum	DFLac	0.0000012	0.0000062	0.849	0.906	-0.00001095	0.00001335
Bifidobacterium longum	3’SL	0.0000536	0.0000156	0.003	0.071	0.00002302	0.00008418
Bifidobacterium longum	6’SL	-0.0000253	0.0000176	0.167	0.399	-0.00005980	0.00000920
Bifidobacterium longum	LNT	-0.0000014	0.0000076	0.853	0.906	-0.00001630	0.00001350
Bifidobacterium longum	LNnT	0.0000292	0.0000230	0.219	0.399	-0.00001588	0.00007428
Bifidobacterium longum	LNFPI	-0.0000008	0.0000069	0.906	0.906	-0.00001432	0.00001272
Bifidobacterium longum	LNFPII	0.0000039	0.0000086	0.650	0.856	-0.00001296	0.00002076
Bifidobacterium longum	LNFPIII	-0.0007846	0.0002766	0.011	0.071	-0.00132674	-0.00024246
Bifidobacterium longum	LSTb	0.0001164	0.0000566	0.054	0.241	0.00000546	0.00022734
Bifidobacterium longum	LSTc	-0.0000697	0.0000466	0.151	0.399	-0.00016104	0.00002164
Bifidobacterium longum	DFLNT	-0.0000129	0.0000045	0.010	0.071	-0.00002172	-0.00000408
Bifidobacterium longum	LNH	0.0000421	0.0000344	0.236	0.399	-0.00002532	0.00010952

Bifidobacterium longum	DSLNT	0.0000495	0.0000300	0.115	0.346	-0.00000930	0.00010830
Bifidobacterium longum	FLNH	-0.0000067	0.0000170	0.697	0.856	-0.00004002	0.00002662
Bifidobacterium longum	DFLNH	-0.0000113	0.0000165	0.501	0.751	-0.00004364	0.00002104
Bifidobacterium longum	FDSLNH	-0.0000197	0.0000109	0.086	0.330	-0.00004106	0.00000166
Bifidobacterium longum	DSLNH	0.0001030	0.0000332	0.006	0.071	0.00003793	0.00016807
Bifidobacterium longum	Delivery mode: emergency caesarean	4.5786764	1.9291254	0.028	0.153	0.79759062	8.35976218
Bifidobacterium longum	Delivery mode: planned caesarean	0.5165475	1.2495966	0.684	0.856	-1.93266184	2.96575684
Bifidobacterium longum	First week pacifier: yes	-0.8715254	1.3563720	0.528	0.751	-3.53001452	1.78696372
Bifidobacterium longum	Siblings: yes	2.1275411	1.2342009	0.101	0.341	-0.29149266	4.54657486
Bifidobacterium longum	Pre-pregnancy BMI: high and obese	-1.4983366	1.2031084	0.228	0.399	-3.85642906	0.85975586
Lactobacillus gasseri	2'FL	0.0000000	0.0000029	0.993	0.993	-0.00000568	0.00000568
Lactobacillus gasseri	3FL	0.0000069	0.0000070	0.338	0.962	-0.00000682	0.00002062
Lactobacillus gasseri	DFLac	0.0000050	0.0000116	0.672	0.962	-0.00001774	0.00002774
Lactobacillus gasseri	3'SL	0.0000054	0.0000292	0.854	0.962	-0.00005183	0.00006263
Lactobacillus gasseri	6'SL	-0.0000185	0.0000329	0.581	0.962	-0.00008298	0.00004598
Lactobacillus gasseri	LNT	-0.0000040	0.0000142	0.781	0.962	-0.00003183	0.00002383

Lactobacillus gasseri	LNnT	0.0000448	0.0000429	0.310	0.962	-0.00003928	0.00012888
Lactobacillus gasseri	LNFPI	0.0000218	0.0000128	0.106	0.962	-0.00000329	0.00004689
Lactobacillus gasseri	LNFPII	0.0000015	0.0000160	0.926	0.962	-0.00002986	0.00003286
Lactobacillus gasseri	LNFPIII	-0.0011175	0.0005164	0.043	0.962	-0.00212964	-0.00010536
Lactobacillus gasseri	LSTb	0.0000164	0.0001056	0.879	0.962	-0.00019058	0.00022338
Lactobacillus gasseri	LSTc	0.0000230	0.0000870	0.794	0.962	-0.00014752	0.00019352
Lactobacillus gasseri	DFLNT	-0.0000032	0.0000084	0.707	0.962	-0.00001966	0.00001326
Lactobacillus gasseri	LNH	-0.0000935	0.0000643	0.162	0.962	-0.00021953	0.00003253
Lactobacillus gasseri	DSLNT	0.0000332	0.0000560	0.560	0.962	-0.00007656	0.00014296
Lactobacillus gasseri	FLNH	0.0000206	0.0000317	0.523	0.962	-0.00004153	0.00008273
Lactobacillus gasseri	DFLNH	-0.0000172	0.0000309	0.584	0.962	-0.00007776	0.00004336
Lactobacillus gasseri	FDSLNH	0.0000021	0.0000203	0.920	0.962	-0.00003769	0.00004189
Lactobacillus gasseri	DSLNH	-0.0000101	0.0000620	0.872	0.962	-0.00013162	0.00011142
Lactobacillus gasseri	Delivery mode: emergency caesarean	3.8589445	3.6015373	0.297	0.962	-3.20006861	10.91795761
Lactobacillus gasseri	Delivery mode: planned caesarean	-1.7949999	2.3329064	0.451	0.962	-6.36749644	2.77749664
Lactobacillus gasseri	First week pacifier: yes	-1.0129822	2.5322483	0.694	0.962	-5.97618887	3.95022447

Lactobacillus gasseri	Siblings: yes	-2.3429957	2.3041638	0.322	0.962	-6.8591567 5	2.1731653 5
Lactobacillus gasseri	Pre-pregnancy BMI: high and obese	-1.6014795	2.2461161	0.485	0.962	-6.0038670 6	2.8009080 6
Porphyromonas sp.	2’FL	-0.0000020	0.0000023	0.399	0.859	-0.0000065 1	0.0000025 1
Porphyromonas sp.	3FL	-0.0000091	0.0000055	0.112	0.756	-0.0000198 8	0.0000016 8
Porphyromonas sp.	DFLac	0.0000091	0.0000091	0.328	0.859	-0.0000087 4	0.0000269 4
Porphyromonas sp.	3’SL	-0.0000058	0.0000228	0.800	0.864	-0.0000504 9	0.0000388 9
Porphyromonas sp.	6’SL	0.0000121	0.0000257	0.644	0.859	-0.0000382 7	0.0000624 7
Porphyromonas sp.	LNT	-0.0000142	0.0000111	0.215	0.859	-0.0000359 6	0.0000075 6
Porphyromonas sp.	LNnT	-0.0000272	0.0000335	0.428	0.859	-0.0000928 6	0.0000384 6
Porphyromonas sp.	LNFPI	0.0000012	0.0000100	0.904	0.904	-0.0000184 0	0.0000208 0
Porphyromonas sp.	LNFPII	0.0000219	0.0000125	0.095	0.756	-0.0000026 0	0.0000464 0
Porphyromonas sp.	LNFPIII	0.0007304	0.0004032	0.086	0.756	-0.0000598 7	0.0015206 7
Porphyromonas sp.	LSTb	0.0000523	0.0000825	0.534	0.859	-0.0001094 0	0.0002140 0
Porphyromonas sp.	LSTc	0.0000622	0.0000679	0.371	0.859	-0.0000708 8	0.0001952 8
Porphyromonas sp.	DFLNT	0.0000024	0.0000066	0.719	0.859	-0.0000105 4	0.0000153 4
Porphyromonas sp.	LNH	-0.0000277	0.0000502	0.588	0.859	-0.0001260 9	0.0000706 9
Porphyromonas sp.	DSLNT	-0.0001011	0.0000438	0.032	0.756	-0.0001869 5	-0.0000152 5

Porphyromonas sp.	FLNH	0.0000277	0.0000248	0.278	0.859	-0.0000209 1	0.0000763 1
Porphyromonas sp.	DFLNH	-0.0000159	0.0000241	0.518	0.859	-0.0000631 4	0.0000313 4
Porphyromonas sp.	FDSLNH	-0.0000048	0.0000158	0.763	0.859	-0.0000357 7	0.0000261 7
Porphyromonas sp.	DSLNH	-0.0000154	0.0000484	0.754	0.859	-0.0001102 6	0.0000794 6
Porphyromonas sp.	Delivery mode: emergency caesarean	-3.1429849	2.8121370	0.278	0.859	-8.6547734 2	2.3688036 2
Porphyromonas sp.	Delivery mode: planned caesarean	0.5857516	1.8215700	0.751	0.859	-2.9845256 0	4.1560288 0
Porphyromonas sp.	First week pacifier: yes	-1.4801732	1.9772193	0.463	0.859	-5.3555230 3	2.3951766 3
Porphyromonas sp.	Siblings: yes	-0.9658253	1.7991273	0.598	0.859	-4.4921148 1	2.5604642 1
Porphyromonas sp.	Pre-pregnancy BMI: high and obese	-1.6135395	1.7538028	0.369	0.859	-5.0509929 9	1.8239139 9

2'-fucosyllactose (2'FL), 3'-fucosyllactose (3FL), difucosyllactose (DFLac), 3'-sialyllactose (3'SL), 6'-sialyllactose (6'SL), difucosyl-N-hexosyl (DFLNH), difucosyl-N-acetyl (DFLNT), disialyl-N-hexosyl (DSLNH), disialyl-N-acetyl (DSLNT), fucosyl-disialyl-N-hexosyl (FDSLNH), fucosyllacto-N-hexaose (FLNH), lacto-N-fucopentaose I (LNFP I), lacto-N-fucopentaose II (LNFP II), lacto-N-fucopentaose III (LNFP III), lacto-N-hexaose (LNH), lacto-N-neotetraose (LNnT), lacto-N-tetraose (LNT), lacto-N-neotriose (LSTc), lacto-N-neotriose (LSTb).
